# Global dynamics of functional composition in CITES‐traded reptiles

**DOI:** 10.1002/eap.3060

**Published:** 2024-11-20

**Authors:** Dominic Meeks, Oscar Morton, David P. Edwards

**Affiliations:** ^1^ Ecology and Evolutionary Biology School of Biosciences, University of Sheffield Sheffield UK; ^2^ Department of Plant Sciences University of Cambridge Cambridge UK

**Keywords:** CITES, ecological function, functional diversity, functional traits, reptile trade, targeted management, wildlife trade

## Abstract

Global wildlife trade is a billion‐dollar industry, with millions of individuals traded annually from a diversity of taxa, many of which are directly threatened by trade. Reptiles exhibiting desirable life‐history or aesthetic traits, such as large body sizes or colorful morphologies, are traded preferentially. A key issue is understanding geographic and temporal variation between desirable species traits and their trade. Poor understanding of this can generalize patterns of consumer trait preferences and conceal functional consequences of wild harvest in ecosystems. Using records of legal, international trade in Convention on International Trade in Endangered Species (CITES)‐listed reptiles between 2000 and 2020, we examine geographic and temporal variation in the functional composition of traded assemblages, both captive‐ and wild‐sourced, identifying key hotspots and routes of functional diversity in trade. We also identify associations between functional traits and species presence in trade. We find that functionally diverse trade assemblages are exported primarily from the tropics, with hotspots in sub‐Saharan Africa, and imported across Asia, Europe, and North America. Patterns of functional composition in trade remained broadly stable from 2000 to 2020. Globally, the species most likely to be traded were large, fecund, generalists. Sustained wild harvest of functionally diverse reptilian assemblages in trade hotspots, such as Madagascar and Indonesia, places substantial pressure on large‐bodied reptiles that fulfill important ecological functions, including population control and nutrient cycling, while also endangering harvest‐vulnerable species with slow life histories. Despite limited species‐specific descriptions of reptilian ecological functions, management in harvest hotspots can safeguard ecosystem functioning by prioritizing protection for threatened species that contribute disproportionately to local and regional functional diversity.

## INTRODUCTION

The legal wildlife trade is a lucrative global industry, moving over 100 million animal and plant products per year with an estimated annual worth of ~US $300 billion (Harfoot et al., 2018; Smith et al., 2017). Species are utilized for a range of reasons, including as pets, food products, traditional medicines, and leather for fashion items (TRAFFIC, 2008). Anthropogenic demand for wildlife products creates a financial incentive to rapidly exploit wild populations, which remain the primary source of wildlife products for many taxa despite the growth of captive breeding, with 22,770,550 wild‐sourced reptiles, 3,220,816 wild‐sourced mammals, and 1,328,474 wild‐sourced birds exported between 2005 and 2014 (Harfoot et al., 2018). Estimates posit that 24% of all terrestrial vertebrates are traded in some form, compounding existing pressures and contributing to elevated species extinction risk and population declines among some traded species (Marsh et al., 2022; Morton et al., 2021; Scheffers et al., 2019). Harvest pressure on wild populations is projected to increase in future decades, with human population growth concentrated in biodiversity hotspots (Hughes, Auliya, et al., 2023).

Traded species are not randomly distributed across the tree of life, with a variety of functional traits influencing the presence and abundance of species within the wildlife trade for different purposes. For example, the prevalence of large‐bodied species in trade is described at local and global scales and attributed to a greater return on hunting effort (Jerozolimski & Peres, 2003). Existing literature outlines the complexities that underlie species selection and functional traits within wildlife trade (Hughes, Auliya, et al., 2023). Among birds, functional and aesthetic traits are associated with trade, with large body mass, distinctive plumage, ability to talk, and high‐quality song being the strongest predictors of trade probability and price (Hughes, Auliya, et al., 2023; Yin et al., 2020). Morton et al. (2023) also highlight divergent associations in reproductive traits between wild‐sourced and captive‐bred assemblages, with captive trade volume greatest among longer lived and early maturing species, and wild‐caught trade volume poorly associated with life‐history traits. More broadly, Street et al. (2023) identify distinct life‐history profiles between vertebrate species traded for pets and products, with pets exhibiting longer, slower reproductive lifespans than product species.

Variation within functional traits underpins species' roles in ecosystems and often their responses to anthropogenic pressures. Multifaceted interactions between biological traits, including body mass, clutch size, diet, age at maturity, and range size, produce divergent responses to climate change, land‐use change, and trade across taxa (Laureto et al., 2015; Rodríguez‐Caro et al., 2023). Functional diversity metrics help understand these complex trait interactions by creating a multidimensional functional space that quantifies the combinations of functional trait values exhibited by all species within a community (Mouillot et al., 2013). Functional diversity metrics have been utilized to identify trait combinations of birds and mammals with the greatest vulnerability to climate change and project how future turnover within these assemblages will affect ecological functioning (Bender et al., 2019). Predicted extinctions of threatened species are projected to force the homogenization of ecological strategies of global avian, mammalian, and reptilian communities toward small‐bodied, fast‐lived, highly fecund generalists (Cooke et al., 2019; Rodríguez‐Caro et al., 2023). Functional homogenization threatens the provision of key ecological functions, such as seed dispersal, nutrient cycling, predator control, and pollination, which underpin ecosystem processes and maintain ecological integrity (Valiente‐Banuet et al., 2015). A key research frontier when we consider the global wildlife trade is how the functional composition of traded assemblages varies across space and time at global, continental, and national scales (Hughes, Auliya, et al., 2023).

Here, we investigate spatiotemporal variation in functional composition within reptile trade. Between 1975 and 2014, ~152 million reptile specimens were traded internationally, with the wider trade involving ~36% of described reptile species (Harfoot et al., 2018; Marshall et al., 2020). We utilize trade data collected by the Convention on International Trade in Endangered Species (CITES) of Wild Fauna and Flora, which documents legal, international trade in CITES‐listed species (CITES, 2022). We tackle four core objectives: (1) identify exporting and importing hotspots of functionally diverse trade; (2) explore the functional composition of different traded reptile assemblages; (3) explore how the functional composition of traded assemblages varies through time; and (4) assess associations between functional traits and trade probability and trade volume among CITES‐listed reptiles at a global and continental scale.

## METHODS

### Data collection

Current CITES listings of reptile species from Appendices I, II, and III were accessed from https://checklist.cites.org/#/en. Trade volumes were calculated from the complete CITES trade database (version 2022.1), which contains >23 million directional records of legal international trade in Listed reptile species since 1975. We focused on comparative exporter‐reported trade records for reptiles between 2000 and 2020, with reexports removed from the dataset. We acknowledge that exporter‐reported trade can sometimes be based on issued permits, inflating trade volumes above the true values; however, as importing countries are not required under CITES to issue permits for (and report) trade involving Appendix II species, we choose to use exporter‐reported data to ensure we capture the full scope of CITES reptile trade (Robinson & Sinovas, 2018). Following previous studies using CITES data (Harfoot et al., 2018; Morton et al., 2022, 2023), we also reproduce our four main figures with importer‐reported data for comparison (Appendix S1: Figures S14–S17). Crucially, our key findings remain supported in both datasets. We utilized wild‐sourced records (those with the source code W, X, and R) and captive‐sourced records (source code C, D, and F) of species traded for commercial purposes (defined as purpose code P or T) (Bush et al., 2014). Inconsistencies or laundering within CITES reporting may result in the mislabeling of farmed specimens as wild‐sourced, but as this issue has not been reported at a large scale and remains challenging to detect, it is unlikely to change our findings (Morton et al., 2024; Schlaepfer et al., 2005). As records are traded in numerous terms and units, all records were converted to whole organism equivalents (WOE's; as per Harfoot et al., 2018) to allow volume comparisons.

Functional traits were accessed from a published dataset containing trait values for described reptile species (Etard et al., 2020a, 2020b). As traits underpin functional diversity metrics, their relevance to ecological functioning and the trade selection process was imperative. Four traits were used: body mass, clutch size, maximum longevity, and habitat breadth (see Appendix S1: Table S1 for trait justifications). Aesthetic traits were not considered due to the lack of a unified coloration dataset for reptiles.

### Data preparation

The CITES trade dataset should only reliably be used up to 2020 to reflect that some Parties will not have yet submitted their most recent reports; therefore, any species Listed after 2020 were removed from the dataset (*n* = 56), reducing the number of Listed species present in our dataset from 1181 to 1125. The availability of functional trait data varied between our traits among the remaining 1125 species (Initial percentage coverage per trait—maximum longevity: 68.5%, habitat breadth: 60.5%, body mass: 99.4%, clutch size 88.9%). We used the IUCN “rredlist” package and the rl_habitats() function to recalculate all species' habitat breadth (Chamberlain, 2020) and conducted a literature review to source missing trait values of individual species (Appendix S1: Table S2), with trait coverage improved following these additions (maximum longevity: 74.8%, habitat breadth 76.2%, body mass: 100% clutch size: 91.7%) To impute the remaining data, we considered using genus and family averages or K‐nearest neighbor (KNN) approaches but opted to utilize a multivariate imputation by chained equations (MICE) approach without phylogenetic information utilizing the “mice” package (van Buuren & Groothuis‐Oudshoorn, 2011). This choice was informed by Penone et al. (2014), which found that the MICE performed better than KNN and MICE approaches that utilized phylogenetic information when imputing life‐history traits. We reproduced the main‐text figures by randomly selecting and utilizing one of the four alternative imputed datasets from the MICE imputation for missing functional trait values (Appendix S1: Figures S18–S21). Following imputation, we had data available for the 295 traded species and 830 non‐traded species. Non‐traded species, some of which are locally harvested for subsistence, included Ruibal's least gecko (*Sphaerodactylus ruibali*) and Cochin forest cane turtle (*Vijayachelys silvatica*), which exhibit restricted ranges and discrete behavioral activity making wild harvest unfavorable and restricting international demand (IUCN, 2024).

For our overall objective—to analyze the functional composition of traded CITES‐Listed reptile assemblages—we created a dataset containing trade volumes and trait values for all Listed species. Non‐traded Listed species were assigned zero values for trade volume in each continent per year. Documentation of species' occurrence or absence on individual continents was incorporated into datasets to improve inferences regarding exported trade assemblages. Twenty‐six species had mismatched names and were updated accordingly. In the trade dataset, nine species were removed from CITES listings between 2000 and 2020 and were subsequently removed from our datasets in the years following their respective removals. Following these amendments, the trade and trait datasets were merged to generate our trade matrices.

### Data analysis

All data analysis and visualization were conducted in R version 4.2.3 (R Core Team, 2023).

We use “assemblages” as a general term to refer to subsets of traded species, for example, the subset of species exported from Africa in 2005.

#### Functional composition of hotspots and trade routes

We created matrices for the total trade assemblages between 2000 and 2020 for exports, imports, and trade routes at a national and continental scale (the structure of which is elucidated in Appendix S1: Table S3), as well as matrices for continental trade routes in each year of trade (trade routes describe links between a specified exporter and importer). To capture the complexity of global reptile trade, we also explored how functional diversity metrics varied between different sources (wild‐sourced and captive‐bred), markets (live and dead specimens—a proxy measure of trade for pets versus trade for leather, meat, and medicine, respectively), and threat status' (we use IUCN classifications of extant species to create broad threat categorizations—Threatened: Critically Endangered, Endangered, and Vulnerable; Non‐threatened: Near Threatened and Least Concern; Data Deficient species were not included) across time and space.

To elucidate the functional composition of traded assemblages, we calculated the functional richness (FRic) and functional specialization (FSpe) values of national and continental traded assemblages of wild‐sourced Listed reptiles. FRic quantifies the volume (range of values of multiple functional traits) occupied by a specific assemblage of species within a multidimensional functional space, which describes the total range of functional trait values of an overall species pool. In our study, FRic quantifies the range of functional trait values exhibited by the species of traded assemblages, in the context of a functional space composed of all Listed reptiles, helping to identify assemblages containing species that exhibit distinct combinations of functional trait values. We also identify trade assemblages where species exhibiting extreme functional trait values, such as the Aldabra giant tortoise (*Aldabrachelys gigantea*) with a maximum longevity value of 180 years, were traded in relatively high abundance. We considered utilizing functional divergence, but this metric measures the abundance of specimens with extreme traits from the center of each individual assemblage, making comparisons between assemblages challenging. Functional evenness, which measures the regularity of abundance distribution within an assemblage, was also considered, but this metric would not distinguish whether extreme traits or traits closer to the center of the functional space were traded in abundance. We selected FSpe as it produces an abundance‐weighted measure of the mean distance to the centroid of the functional space, providing us with a metric to compare the relative prominence of species with extreme functional trait values between traded assemblages (Mouillot et al., 2013). We define hotspots as countries with national trade assemblages (exported or imported) values in the top five percent of values for the specified functional metric.

Prior to analysis, body mass, clutch size, and maximum longevity were transformed by log10, and all traits were standardized to zero mean and unit variance (z‐transformation used for variables with different measurement scales) (Villéger et al., 2008). We utilized the functions tr.cont.fspace() and alpha.fd.multidim() in the “mFD” package to generate the functional space of all Listed reptiles and the FRic and FSpe values for each assemblage (Magneville et al., 2022). The princomp() and funspace() functions in the “funspace” package were used to visualize the functional space of various traded assemblages (Carmona et al., 2024). We reduced the number of functional axes from four to three for our analysis as three principal components described 93.93% of all variation in the functional space and the lowest mean squared error. For functional diversity metrics to be generated, trade assemblages must contain at least one species more than the number of principal components. Accordingly, we excluded all countries that exported or imported three or fewer species from our trade matrices, accounting for 51.04% of the 96 exporting countries and 33.56% of the 146 importing countries. Despite representing a high percentage of countries, the impact of these exclusions is limited, with the combined trade volume of excluded countries only representing 0.48% of the total trade volume of all assemblages.

Standard effect size functional richness (SES FRic) values were utilized to determine whether FRic values of traded assemblages were greater or less than expected considering their species richness (Swenson, 2014).

SES FRic values were generated using the following formula:

$$\mathrm{SES}\;\text{FRic}=\frac{\text{FRic of traded assemblage}-\text{mean FRic of random assemblages}}{\mathrm{SD}\;\text{of FRic from random assemblages}}$$

Random assemblages were created by randomly selecting Listed reptile species (all species that could potentially be recorded in trade) from our overall traded and non‐traded dataset in a quantity equivalent to the number of species present within each traded assemblage. We randomly assigned the trade volumes present within the traded community to the species present in the random assemblage. FRic values were obtained from each of the 999 random assemblages, with the mean and SD values being calculated and input into the formula to produce SES FRic values.

We consider SES FRic values greater than +2 and lower than −2 to exhibit significant deviation from the expectation (Botta‐Dukát, 2018). Assemblages with under‐dispersed (negative) SES FRic values occupy a smaller volume of the trait space than expected, indicating selective preference for species with specific trait values (species with large body mass favored to species with small body mass). Conversely, assemblages with nonsignificant or over‐dispersed (positive) SES FRic values occupy a larger volume of the trait space than expected, indicating random assembly and the absence of filtering for specific functional trait values is absent. Positive SES FRic values may also reflect divergent selection resulting in higher trait variability than expected within a specific regional pool.

#### Functional trait associations with trade

To investigate whether specific functional trait values are associated with the probability of trade and trade volume among Listed reptiles, we first modeled the presence of Listed species in trade relative to the species functional trait values, assuming a Bernoulli error distribution. Secondly, we modeled the trade volume of Listed species when they are traded (e.g., when trade volume >0) relative to the species' functional trait values, assuming a negative binomial error distribution. The functional traits modeled were log (base10) body mass, log clutch size, log maximum longevity, and habitat breadth, with each being standardized prior to modeling and each modeled separately. As we were primarily interested in the association of functional trait values with the probability of trade or volume of trade, we included trait values as a fixed effect and allowed this to vary across years and taxon by including these variables as hierarchical effects. Year was included as a continuous fixed effect and as a factor variable, supplied as a hierarchical varying intercept, to account for both volume trends through time and discrete global fluctuations.

We also modeled the presence of Listed species in trade relative to the species' functional trait values, and the continent from which the species had been exported or imported. Bernoulli error distribution was assumed for these models. As we were primarily interested in the association of trait values with the probability of trade between different continents, we included the trait values interaction with the exporting or importing continent as a fixed effect and allowed this to vary across years and taxon by including these variables as hierarchical effects. The interaction between year and the exporting or importing continent was included as a control fixed effect. To aid interpretation, we divided the coefficients by the SD of the log to base 10 values of the modeled trait (habitat breadth values were not logged), this moved them from the change in log odds per 1 − SD increase to the change in log odds per increase in magnitude assuming all other covariates remain fixed. All models were run for 1000 iterations with 500 warm‐up iterations and for four chains. Priors were specified as zero‐centered and regularizing for all model parameters.

All models were checked to ensure appropriate fit and convergence. Models were visually assessed to ensure chain mixing and stable convergence. All Rhat (potential scale reduction factor) values were checked to be <1.05, indicating both between and within chain estimates had converged. Posterior predictive checks were used to assess individual model adequacy and check for systemic discrepancies between features of the real and simulated data.

All data manipulation was conducted using the “tidyverse” ecosystem of packages (Wickham et al., 2019). Functional space quantification and functional metric calculation were conducted with “mFD” (Mouillot et al., 2013). Functional diversity metrics were mapped using packages rnaturalearth, sf, countrycode, ggplot2 and ggpubr (Arel‐Bundock et al., 2018; Kassambara, 2023; Massicotte et al., 2023; Pebesma, 2018; Wickham, 2016). Functional metrics of trade routes were plotted using package networkD3 (Allaire et al., 2017). All model fitting, testing, and interpretation were conducted using “brms” (Bürkner, 2017) and “tidybayes” (Kay, 2023).

## RESULTS

### Functional patterns of global reptile trade

#### Geographic distribution of trade hotspots

**FIGURE 1:**
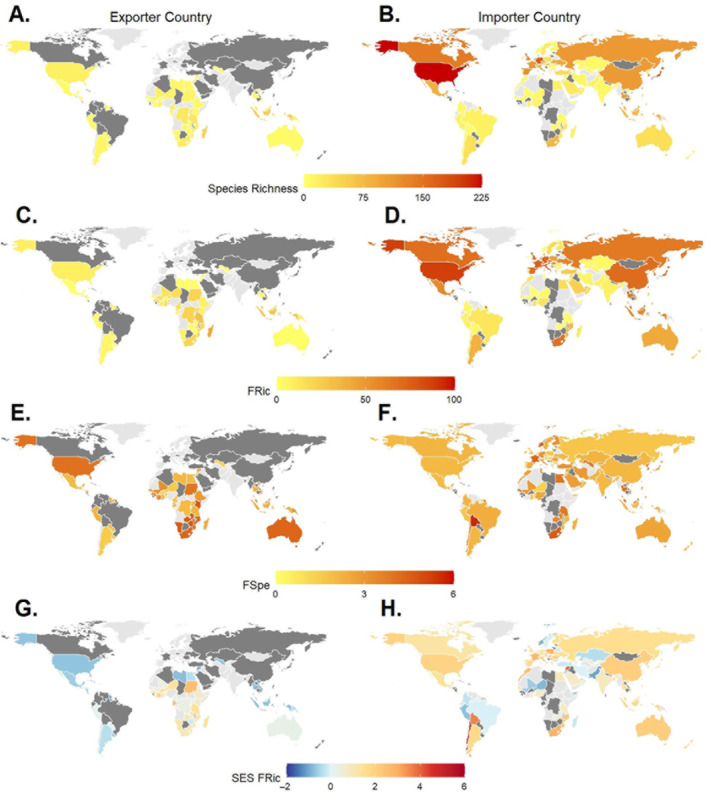
Species richness values of national (A) exported and (B) imported assemblages. Functional richness (FRic) values of national (C) exported and (D) imported assemblages. Functional specialization (FSpe) values of national (E) exported and (F) imported assemblages. Standard effect size (SES) FRic values of national (G) exported and (H) imported assemblages. Countries shaded in light gray did not document any trade between 2000 and 2020, while countries shaded in dark gray did not export or import enough species to generate functional metrics.

Total exported assemblages from Africa and Asia exhibited the greatest continental‐level FRic (Africa [60.96], Asia [28.29]) (Appendix S1: Figure S1). Of the top 10 national exported assemblages with the highest FRic values, eight originated from African countries, the top two being Madagascar and Tanzania, with Indonesia and Malaysia contributing the remaining two assemblages (Figure 1). National and continental exported assemblages of Africa and Asia also exhibit the highest values of FRic for threatened trade assemblages (FRic: Africa [43.88], Asia [13.11]; national assemblages: Appendix S1: Figure S2). Live African exported assemblages were more speciose and made a greater contribution to the continents' overall FRic value than African dead exported assemblages (FRic: live‐traded [52.41], dead‐traded [22.69]; Appendix S1: Figure S3). The FRic values of European (72.11), North American (70.32), and Asian (67.05) imported assemblages exceeded values exported by any continent. The 10 national imported assemblages with the highest FRic values were composed of the United States, five European countries, and four Asian countries. Live‐imported assemblages were more speciose than dead‐imported assemblages and made greater contributions to the FRic value of each continental imported assemblage (Appendix S1: Figure S4).

Total exported assemblages of North America and Africa exhibited the greatest continental‐level FSpe (North America [3.35], Africa [2.98]). The high FSpe of these assemblages is due to the substantial export of dead‐traded crocodilians, with 5,999,956 American alligators (*Alligator mississippiensis*) exported from the United States and 1,697,670 Nile crocodiles (*Crocodylus niloticus*) exported by countries across Africa. Total imported assemblages of Africa, Europe, and Asia exhibited the greatest FSpe (Africa [3.43], Europe [3.09], Asia [2.67]).

The SES FRic values of exported continental and national assemblages in Africa were positive but not significantly greater than expected, except Gabon and Libya, which were significantly over‐dispersed but only exported five and four species, respectively (continental assemblage: +0.58 SDs from expected value, Appendix S1: Figure S1G; national assemblages: Figure 1G). Conversely, exported continental and national assemblages in Asia were under‐dispersed but not significantly (continental assemblage: −1.24 SDs; national assemblages: Figure 1G). For imported national assemblages, seven countries exhibited significantly over‐dispersed SES FRic values (total countries = 95), with a further 25 countries exhibiting SES Fric values between +1 and +1.99, while no countries exhibited significantly under‐dispersed values. The imported assemblages of Chile and Qatar exhibit the greatest SES FRic values (Figure 1H); however, with each country importing fewer than five species, this is attributed to a few species with divergent functional trait values (Chile's imported assemblage includes Nile crocodile, panther chameleon [*Furcifer pardalis*], and alligator snapping turtle [*Macrochelys temminckii*]). The FRic values of live‐imported and dead‐imported assemblages of North America, Europe, and Asia did not differ significantly from the expectation (Appendix S1: Figure S4).

The five assemblages of continental trade routes (total routes = 25) with the highest FRic values all originated from Africa (Appendix S1: Figure S5A). Among national route assemblages, the 20 routes with the highest FRic values (total routes = 406) were exported from either Madagascar or Tanzania and imported by Asian, European, or North American countries (Appendix S1: Figure S6). The five assemblages of continental trade routes with the highest FSpe values included routes from North America and Africa to Asia, Europe, and South America (Appendix S1: Figure S5B).

#### Functional composition of CITES trade

**FIGURE 2:**
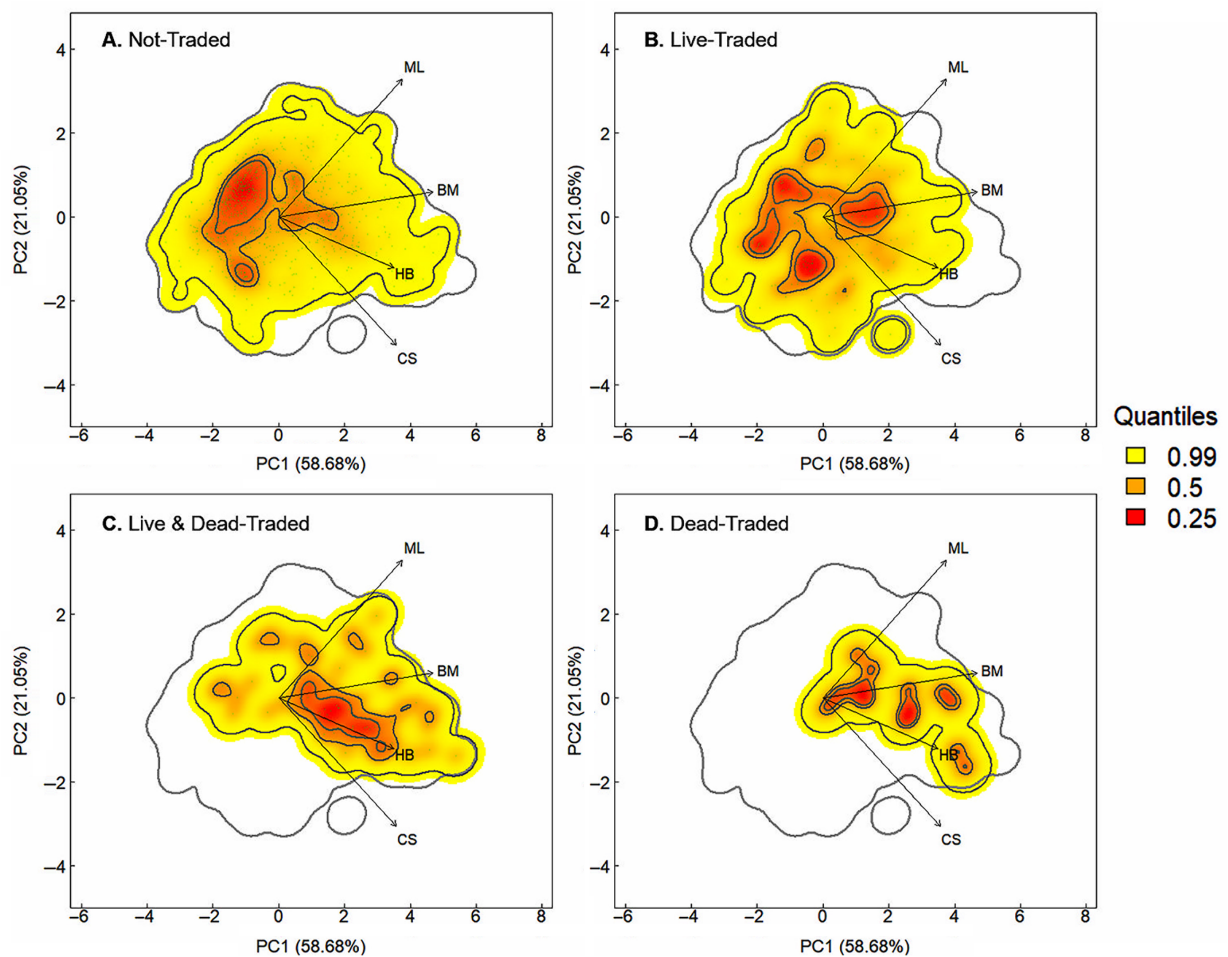
The probabilistic species distribution of (A) non‐traded, (B) dead‐only traded, (C) live and dead‐traded, and (D) live‐only traded assemblages within the functional space of all Convention on International Trade in Endangered Species (CITES)‐listed reptile species, defined by the two first principal components axes (PC1 = 58.68% and PC2 = 21.05% of variance explained) of a principal components analysis (PCA). The arrows indicate the direction and weighting of the functional traits (body mass [BM], clutch size [CS], maximum longevity [ML], habitat breadth [HB]) in the PCA. The color gradient (red, orange, and yellow) depicts the density of species in the functional space, where red corresponds to more densely populated areas. Gray contour lines indicate the outer limits of the functional space and black lines indicate quantiles 0.25, 0.5, 0.99.

We identify distinct functional differences between live‐traded and dead‐traded assemblages. The live‐only trade assemblage occupies a large region of the overall CITES‐listed functional space (FRic = 41.03), similar in coverage to the non‐traded assemblage, and exhibits regions of high species density (peaks) around lower‐than‐median values of all functional traits (Figure 2A,B). Conversely, dead‐only and live‐and‐dead trade assemblages occupy a comparatively smaller region of the functional space (FRic: dead‐only [24.09], live‐and‐dead [32.38]), with both exhibiting distinct peaks around higher‐than‐median values of body mass and habitat breadth (Figure 2C,D). Wild‐only and captive‐only assemblages exhibit distinct functional compositions despite occupying similar volumes of the functional space (FRic wild‐only [27.54], captive‐only [29.18]), with wild‐only assemblages peak around lower‐than‐median body mass values and captive‐only assemblages peak around median and higher‐than‐median body mass and habitat breadth values (Appendix S1: Figure S7).

### Temporal variation in the functional composition of traded assemblages

**FIGURE 3:**
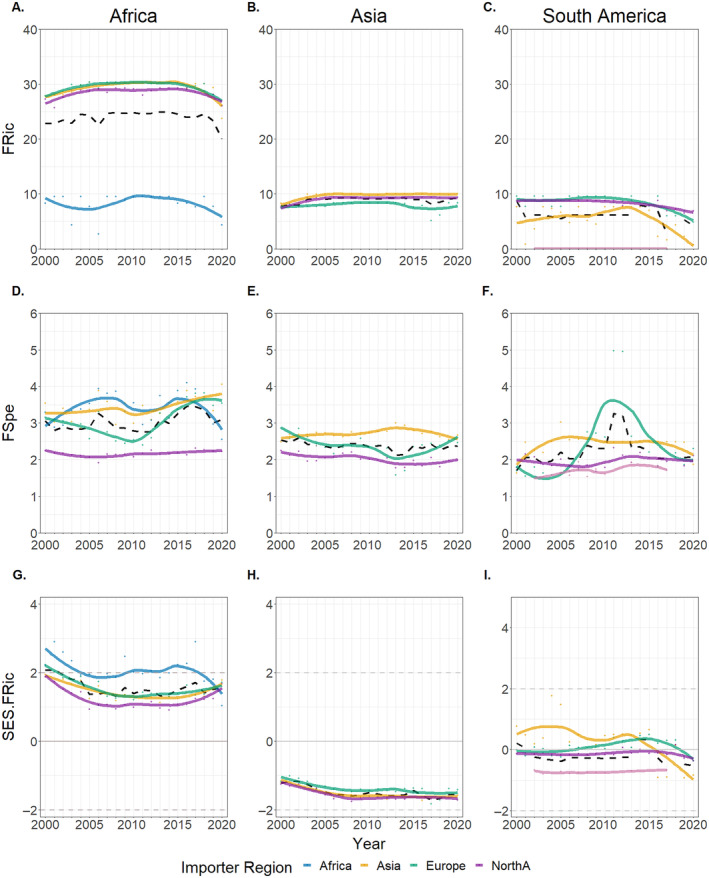
Functional richness (FRic) (A, B, C), Functional specialization (FSpe) (D, E, F), and Standard effect size (SES) FRic (G, H, I) values associated with continental trade routes originating from Africa (A, D, G), Asia (B, E, H), and South America (C, F, I) between 2000 and 2020, with colored lines representing the locally estimated scatterplot smoothing (LOESS) values of each trade route. Dashed black lines represent the 5‐year rolling‐average value and dashed gray lines at +2 and −2 SES FRic values signify (two SDs) a significant departure from expectation.

While we observe differences in the FRic and SES FRic values between assemblages of routes originating from distinct exporters, temporal stability was generally observed across the assemblages of global trade routes. Assemblages of trade routes originating from Africa consistently exhibit the greatest FRic values and the most over‐dispersed SES FRic values of all trade routes between 2000 and 2020. FRic and SES FRic values of assemblages originating from the same exporter exhibited stability throughout 21 years of trade, with the exception of assemblages exported from Asia, which display moderately under‐dispersed SES FRic values that have, on average, declined since 2005 (Figure 3).

We identify temporal stability in the overall trends of FSpe values across global trade routes, with rolling‐average values remaining consistent throughout the 21 years of trade. Despite this overall stability, we also observe a considerable peak of FSpe values in the continental routes from South America to Europe from 2010 to 2014. We assessed species additions and deletions from CITES listings in the years surrounding the observed peaks, with only one newly added species being native to South America, the black caiman (*Melanosuchus niger*), of which only 11 specimens were exported in 2011. Instead, these peaks are likely attributed to substantial fluctuations in the trade of wild‐caught spectacled caiman (*Caiman crocodilus*). Between 2010 and 2013, 116,232 specimens were traded along these trade routes, which fell sharply to only 743 specimens between 2014 and 2020, possibly resulting from an increase in captive breeding or displacement in the reptile skin market by American Alligator exported from the United States.

From 2000 to 2020, the species richness (hereafter referred to as richness) of wild‐sourced and captive‐bred exports diverged, with a steady decline among wild‐sourced exports contrasted by a considerable increase among captive‐bred exports (Appendix S1: Figure S8). Corresponding increases in the FRic and FSpe of captive‐bred exports were not observed, with values remaining roughly equal to those observed for wild‐sourced assemblages across the 21 years of trade despite the drop in wild‐sourced richness. SES FRic values of wild‐sourced exports remained considerably over‐dispersed and greater than the values of captive‐bred exports.

Between 2000 and 2020, sharp increases in the richness and FRic of non‐threatened trade assemblages were observed, owing to the first assessment of many previously unassessed species from the families Gekkonidae and Chamaeleonidae as non‐threatened (Appendix S1: Figure S9). FSpe values were higher among non‐threatened trade assemblages due to greater trade volumes among non‐threatened species than threatened species. Threatened traded assemblage richness increased moderately while FRic and SES FRic values of threatened assemblages increased considerably throughout the 21 years of trade. The FRic of non‐threatened trade was consistently lower than the FRic of total trade, demonstrating that traded threatened species occupied unique positions within the functional space of traded Listed reptiles.

### Functional trait associations with trade

Globally, we find that larger body mass, greater habitat breadth, larger clutch sizes, and longer maximum longevity were associated with an increased probability of trade (Appendix S1: Figure S10, Table S4). Increases in the magnitude of clutch size and habitat breadth were also associated with significant increases in trade volume (Percentage increase in trade volume: clutch size [1.16], habitat breadth [2.13]; Appendix S1: Figure S11, Table S5). While the probability of being traded is greatest among large, highly fecund, generalist species, the overall number of traded species is highest among small, poorly fecund, generalist species, with lower trade probabilities among such species attributed to the high number of Listed species exhibiting small trait values.

**FIGURE 4:**
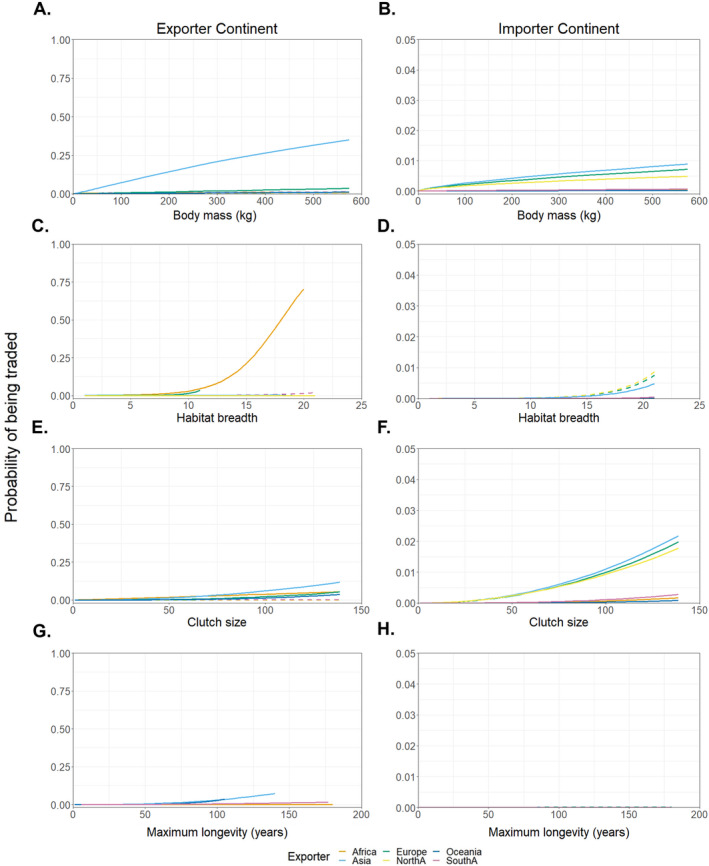
Associations between the probability of being traded and functional traits in exported (A, C, E, G) and imported (B, D, F, H) continental assemblages. Body mass (A, B), habitat breadth (C, D), clutch size (E, F), and maximum longevity (G, H). Lines represent the posterior median value associated with each continent, solid lines show a substantial posterior certainty of direction (e.g., clear positive or negative association classed as >97.5% of the posterior direction sharing sign with the median), and dashed lines show an unclear direction (coefficient values can be found in Appendix S1: Tables S5 and S6).

Among all continental exported assemblages, Asian exports display the strongest positive associations between the probability of trade and body mass and clutch size, while African exports display the strongest and positive association between the probability of trade and habitat breadth (Figure 4A,C,E; Appendix S1: Figure S12, Table S6). The imported assemblages of Asia, Europe, and North America exhibit strong, positive associations between the probability of trade and body mass and clutch size (Figure 4B,F; Appendix S1: Figure S13, Table S7). Conversely, imported assemblages from Africa, Oceania, and South America exhibit weak associations between the probability of trade and each functional trait.

## DISCUSSION

We identified high levels of exported functional diversity from Sub‐Saharan Africa and Southeast Asia. Conversely, the majority of functional diversity flowed into Europe, North America, and Asia. Across exporters and importers, there was a higher prevalence of larger, more generalist, more fecund, and longer lived species. Improved monitoring of global reptile trade and further research to fully assess the ecological consequences of trade and inform targeted conservation efforts are required.

### Functional patterns of global reptile trade

Globally, we find hotspots of functionally diverse exported trade in regions of high biodiversity (Tanzania) and endemism (Madagascar). These regions are also epicenters of legal and illegal trade across taxonomic groups (Cox et al., 2022; Gumbs et al., 2020; Morgan, 2018), with sub‐Saharan Africa and Southeast Asia representing trade hotspots of phylogenetic and functional diversity among mammals and birds (Hughes, Massam, et al., 2023). Our hotspots largely exhibit good agreement with documented global hotspots of trade (Hughes, Auliya, et al., 2023; Scheffers et al., 2019), with divergences, including the absence of Australia as an exporting hotspot in our study, likely due to their stringent national legislation limiting the export of native species. Wider differences likely reflect the Scheffers et al. (2019) species pool of all possibly traded species and the CITES species pool used in our study, which only focuses on internationally traded species. We also find that species richness scales with functional diversity in CITES‐reported trade, with no individual nations exhibiting FRic values that were significantly greater or lesser than expected. South America's absence as a key region of traded FRic, despite representing a global hotspot of reptilian diversity (Roll et al., 2017), highlights that high CITES‐traded FRic is not solely dependent on regional species richness. Compliance with CITES regulation and the strength of illegal trade also represent key factors. For instance, in Brazil, which was excluded from our FRic analysis due to low species count, 19 species of reptiles were identified in TRAFFIC seizures between 2012 and 2019, including at least five species of CITES‐listed river turtles (TRAFFIC, 2020). By integrating multiple sources of trade data (following Marshall et al., 2020), future studies should assess the extent to which the subversion of CITES trade regulation masks hotspots of traded functional diversity.

Hotspots of imported functional diversity identify regions where reptiles are widely utilized. In Asia, chelonians and snakes provide a food resource and are utilized in traditional medicines, while crocodilian and snake leather is used in the fashion industry (Stanford et al., 2020). The E.U. was the top global importer of live reptiles for the pet trade (by value = €7 million) in 2005, while 4.7 million U.S. households own a total of 13.6 million reptiles (Collis & Fenili, 2011; Robinson et al., 2015). Assemblages of live‐traded species, primarily supplying pets, are more speciose and functionally diverse than assemblages of dead‐traded species, likely owing to a selection of nonfunctional traits, such as color, rarity, and unique aesthetic features, which are not strongly correlated with functional traits (Hughes, Auliya, et al., 2023; Stanford et al., 2020). Assemblages of dead‐traded species exhibit substantially greater trade volumes reflecting the scale of the reptile skin industry which is worth an estimated US $1 billion per annum (Kasterine, 2012). Species traded for skins in commercial abundance have greater CITES coverage than those traded in smaller quantities for pets, meaning that while our study may accurately elucidate the functional composition of the global reptile leather trade, our findings regarding the global reptile pet trade may be limited (Marshall et al., 2020).

### Temporal variation in the functional composition of traded assemblages

The temporal stability of FRic values across assemblages of continental trade routes reflects sustained demand for a diversity of reptilian functional trait values in trade to supply both pet and product markets (Robinson et al., 2015). Declining FRic and standardized effect size FRic values were observed in assemblages of trade routes originating from Asia from 2005 to 2020, likely reflecting an increasing selective pressure among Asian wild‐sourced exports for species with functional trait values, including large body mass and high clutch size values, that are desirable for reptile products. Further research is required to determine whether these trends signify a homogenization of functional trait values in Asian traded reptile assemblages or the displacement of wild‐caught trade with captive breeding (Morgan, 2018). Global captive‐bred assemblages exhibited relatively constant FRic values between 2000 and 2020, despite a sharp increase in species richness, highlighting that increases in species diversity do not necessarily equate to higher functional diversity if the functional trait values of newly traded captive‐bred species are not distinct among all traded reptiles. Evaluating whether captive breeding can sustainably displace functionally diverse wild harvests in biodiversity hotspots remains an outstanding research question essential to understanding how the shifting dynamics of specimen sourcing will impact the conservation outcomes of the global reptile trade (Meeks et al., 2024; Robinson et al., 2015).

### Functional trait associations with trade and ecological consequences

Functional trait associations with trade may help forecast the ecological implications of sustained exploitation in biodiversity hotspots. The prevalence of large‐bodied reptiles in international trade mirrors general patterns observed in birds, mammals, and amphibians (Hughes, Massam, et al., 2023; Scheffers et al., 2019), with the highest probability of trade among large reptiles identified in African and Asian exported assemblages. Wild populations of large‐bodied species, such as wild populations of reticulated python (*Malayopython reticulatus*) and Nile crocodile (both Listed), have not experienced detrimental declines in abundance or population characteristics—such as body size—despite substantial annual harvest, owing to large clutch sizes and early age of maturity (Natusch et al., 2016; Thorbjarnarson, 1999). Harvest resilience is not conserved across large‐bodied reptiles, despite ecological similarities, with African rock python (*Python sebae*) and Siamese crocodile suffering substantial trade‐driven population declines (Daltry et al., 2016; VKM et al., 2023). Continued development of functional trait analyses is required to understand divergent species responses to sustained anthropogenic pressure.

Declines among large‐bodied species can alter the provision of ecosystem functions by undermining nutrient cycling, inhibiting plant recruitment, and destabilizing population dynamics across trophic levels (Hughes, Auliya, et al., 2023; Valiente‐Banuet et al., 2015). In the Brazilian Amazon, the extirpation of key large frugivorous mammal species (spider monkey, *Ateles* spp.; woolly monkey, *Lagothrix* spp.; lowland tapir, *Tapirus* spp.), and associated loss of seed dispersal, was predicted to induce an average decline in aboveground biomass of 5.77% with some losses as high as 37.8%. (Peres et al., 2016). With ~470 recorded species of frugivorous lizards, including Gray's monitor (*Varanus olivaceus*), the role of reptiles in maintaining seed dispersal may be considerable, but the provision of this function may be undermined by local, trade‐induced extirpations (Valido & Olesen, 2019). Additionally, reptilian predators, such as the oriental rat snake (*Ptyas mucosa*), can help maintain trophic balance while safeguarding food security and livelihoods in agricultural regions by controlling populations of pest species (Weiss & Kalki, 2023). Trade‐driven declines in the local abundance of predators can provide a competitive release for herbivorous and granivorous species, with consequent increases in grazing intensity potentially limiting food and habitat availability while also undermining plant recruitment in ecosystems (Hughes, Auliya, et al., 2023). De Miranda (2017) also postulates the roles of reptilian herbivores in maintaining trophic balance via prey provision, supporting plant recruitment via the grazing of fast‐growing pioneer species and contributing to seed dispersal networks via frugivorous activity. Research bias toward large, non‐reptile species has restricted species‐specific understanding of reptilian ecological functions; addressing this deficiency represents a key research priority that could help advise conservation prioritization among exploited reptilian populations.

Small, long‐lived, less fecund, specialist species are also present in trade, primarily in the functionally diverse live‐exported assemblages of African and Asian countries (Madagascar and Indonesia), despite low global probabilities of trade. Species that exhibit these traits, including geckos, chameleons, and chelonians, are targeted to provide pets, traditional medicines, and delicacy food items (Stanford et al., 2020). Unlike the reptile skin trade, species selection is driven by aesthetic and cultural factors that inadvertently select for specific functional trait extremes, including low clutch size, associated with slow life histories and increased vulnerability to sustained wild harvest. Corresponding trade‐induced population declines have been observed among African species of tortoise (e.g., ploughshare tortoise [*Astrochelys yniphora*]), geckos, and chameleons as well as Asian chelonian species, with the Yunnan box turtle (*Cuora yunnanensis*) and Burmese star tortoise (*Geochelone platynota*) suffering global and functional extinctions, respectively (Hinsley et al., 2023; Jenkins et al., 2014; Luiselli et al., 2013). Sustained wild harvest of functionally diverse assemblages will compound regional threats, including habitat loss, contributing to reduced local species abundances among harvest‐vulnerable species and potentially compromising ecological functioning in ecosystems within epicenters of sub‐Saharan African and Southeast Asian trade (Atwood et al., 2020).

### Management and conclusions

Our study identifies Madagascar and Tanzania as hotspots of functionally diverse trade and potential regions of compromised ecological functioning; however, limitations of CITES coverage may conceal additional hotspots. CITES trade regulation did not cover ~79% of the 3943 reptile species detected in trade between 2000 and 2019, with 194 of the 355 most threatened species not receiving coverage (Marshall et al., 2020). Key importing regions in Asia and Europe could consider adopting trade documentation systems beyond CITES requirements akin to the United States' Law Enforcement Management Information System (LEMIS), which documents all legal imports of Listed and non‐Listed species (Marshall et al., 2020). Likewise, given the dominance of the global north as an importer, they should bear additional responsibility in ensuring their imports cause no detriment to the exporting Parties' biodiversity.

Our findings emphasize the need for targeted policy and regulation in regions where trade targets species assemblages from across functional space at potentially unsustainable rates. Reptile trade bans have previously produced deleterious conservation outcomes (Mialon et al., 2022), meaning alternative management strategies, including biologically informed, dynamic harvest quotas, should be explored for traded species with slow life‐history trait values, unsuited to sustained harvest (Hughes, Morton, et al., 2023). Management strategies, codeveloped with community members, could encourage harvest during less vulnerable stages of the focal species' life history to prevent deleterious demographic consequences or promote the trade of substitutable species with stable, resilient populations (Natusch et al., 2016; Nossal et al., 2016). Future research could consider a knock‐out approach, evaluating the associated loss of local or regional functional diversity following individual species extirpations, enhancing species conservation prioritization and opening avenues for innovative management strategies. These could include novel payments for ecosystem services schemes to financially incentivize local communities to curtail wild harvest of species with disproportionate contributions to functional diversity (Rodríguez‐Caro et al., 2023; Smith et al., 2011).

Sustained demand for reptiles in Asia, Europe, and North America will continue to drive wild harvest in regions of biodiversity and endemism, such as Madagascar, Tanzania, and Indonesia. Sustained exploitation of species across reptilian functional space threatens the provision of ecological functions in ecosystems surrounding trade epicenters. Failure to enact targeted management strategies may induce irreparable declines and the loss of irreplaceable ecological functions, undermining ecosystem resilience in regions of intense harvest pressure.

## AUTHOR CONTRIBUTIONS

Dominic Meeks and Oscar Morton conceived the ideas and collected the data. Dominic Meeks led the data analysis and writing of the manuscript. All authors contributed critically to the drafts and gave final approval for publication.

## CONFLICT OF INTEREST STATEMENT

The authors declare no conflicts of interest.

## Supporting information


Appendix S1:

## Data Availability

Reptile trait data (Etard et al., 2020a) are available in the file titled “Reptiles.csv” in Figshare at https://doi.org/10.6084/m9.figshare.10075421.v2. The CITES reptile trade dataset (Meeks & Morton, 2023) is available in Figshare at https://doi.org/10.6084/m9.figshare.24593205.
