# Supplementary material for: Global dynamics of functional composition in CITES‐traded reptiles
Source: Ecol Appl. 2024 Nov 20;35(1):e3060. doi: 10.1002/eap.3060 (PMC11733411; doi:10.1002/eap.3060)
Supplement: Supplementary file 1 — Appendix S1: [file EAP-35-e3060-s001.pdf]

Dominic Meeks, Oscar Morton, and David P. Edwards

Global dynamics of functional composition in CITES-traded reptiles

Ecological Applications

Supporting information

Appendix S1: Table S1

Broad and reptile-specific associations between selected traits and ecological function and trade.

Trait	Broad trait association	Trait association within reptiles	References
**Body mass**	Larger body mass values correlate with greater prevalence in trade across all taxa[1]. Larger body mass values are also associated with larger lifespans across a range of vertebrate[2]. In mammals, body mass is negatively correlated with clutch size[3].	In reptiles, larger body mass values are associated with greater lifespans[2] and greater clutch sizes[4].	[1] Scheffers, B. R., Oliveira, B. F., Lamb, I. & Edwards, D.P. (2019). 'Global Wildlife Trade across the Tree of Life'. *Science* 366: 71–76. https://doi.org/10.1126/science.aav5327.<br>[2] Kuparinen, A., Yeung, E. & Hutchings, J.A. (2023) 'Correlation between Body Size and Longevity: New Analysis and Data Covering Six Taxonomic Classes of Vertebrates'. *Acta Oecologica* 119 https://doi.org/10.1016/j.actao.2023.103917.<br>[3]Werner, J., & Griebeler, E. M., (2011). 'Reproductive Biology and Its Impact on Body Size: Comparative Analysis of Mammalian, Avian and Dinosaurian Reproduction'. *PloS One* 6 https://doi.org/10.1371/journal.pone.0028442.<br>[4]Hallmann, K. & Griebeler, E. M. (2018). 'An Exploration of Differences in the Scaling of Life History Traits with Body Mass within Reptiles and between Amniotes'. *Ecology and Evolution* 8 (11): 5480–94. https://doi.org/10.1002/ece3.4069.
**Clutch size**	In birds and mammals, slow life histories are associated with low fecundity[5]. Among mammals and amphibians clutch size is positively	In reptiles, slow life histories are associated with greater fecundity[6], as observed in crocodilians and large monitors (*Varanus sp.*). Among reptiles,	[5]Jeschke, J. M. & Kokko, H. (2009). 'The Roles of Body Size and Phylogeny in Fast and Slow Life Histories'. *Evolutionary Ecology* 23 (6): 867–78. https://doi.org/10.1007/s10682-008-9276-y.<br>[6]Meiri, S., Avila, L., Bauer, A. M., Chapple, D. G., Das, I., Doan, T., Doughty, P., et al. (2020). 'The Global Diversity and Distribution of Lizard Clutch Sizes'. *Global Ecology and*

	associate with probability of trade and frequency of trade[7].	clutch size is positively associated with probability of trade and frequency of trade.	*Biogeography* 29 (9): 1515–30. https://doi.org/10.1111/geb.13124.
**Maximum longevity**	In mammals and amphibians, greater lifetime reproductive output is associated with greater presence in live pet trade[7].	In reptiles, greater lifetime reproductive output is associated with greater probability of trade and frequency of trade among live-traded specimens in the pet trade[7].	[7]Street, S. E., Gutiérrez, J. S., Allen, W. L. & Capellini, I. 2023. 'Human Activities Favour Prolific Life Histories in Both Traded and Introduced Vertebrates'. *Nature Communications* 14 (1): 262. https://doi.org/10.1038/s41467-022-35765-6.
**Habitat breadth**	Broadly, species with narrow environmental niches exhibit small geographic ranges, which are often associated with low local abundances. Ecological specialists exhibit a greater extinction risk from pressures such as overexploitation [8]. Habitat breadth provides a proxy measurement of ecological specialisation	Among reptiles, ecological generalists are anecdotally favoured in trade due to greater return on hunting investment[9, 10], with habitat generalists, *Varanus salvator* and *Python regius*, being among the most abundantly traded reptile species[11]. Despite ecological specialisation potentially dictating both hunting pressure and impact of hunting pressure on local populations, no studies have yet quantified the association between habitat specialisation and	[8]Williams, S. E., Y. M. Williams, J. VanDerWal, J. L. Isaac, L. P. Shoo, and C. N. Johnson. 2009. 'Ecological Specialization and Population Size in a Biodiversity Hotspot: How Rare Species Avoid Extinction'. Proceedings of the National Academy of Sciences 106 (supplement_2): 19737–41. https://doi.org/10.1073/pnas.0901640106. [9]Nossal, K., Mustapha, N., Ithnin, H., Kasterine, A., Khadiejah, S. M.K., Lettoof, D., and Lyons, J.A., and Natusch, D.J.D. 2016. 'Trade in Python Skins: Impact on Livelihoods in Peninsular Malaysia https://intracen.org/resources/publications/trade-in-python-skins-impact-on-livelihoods-in-peninsular-malaysia. [10]Daltry, J., Langelet, E., Solmu, G., Ploeg, J., Weerd, M. & Whitaker, R. (2016). 'Successes and Failures of Crocodile Harvesting Strategies in the Asia Pacific Region'. Tropical Conservation: Perspectives on Local and Global Priorities 21, 345–62. [11]Luiselli, L., Bonnet, X., Rocco, M. & Amori G. (2011). 'Conservation Implications of Rapid Shifts in the Trade of Wild African and Asian Pythons' Biotropica vol. 44, no. 4, 569-573. .

		trade. We aim to address this research gap.	

Appendix S1: Table S2

Data preparation: Missing functional trait values were filled by using values from our literature search. The functional trait, species, and source for the data is provided below.

**Body mass**	
Chelonoidis denticulatus	[1] Regis, K. W. *Allometry of sexual size dimorphism in turtles: A comparison of mass and length data*. https://www.proquest.com/docview/1804413926?fromopenview=true&pq-origsite=gscholar (2016).
**Clutch size**	
Chitra chitra	[2] Bonin, F., Devaux, B. & Dupré, A. *Turtles of the World*. (A & C Black, 2006).
Calumma tarzan	[3] Gehring, P.-S. *et al.* A tarzan yell for conservation: A new chameleon, Calumma tarzan sp. n., proposed as a flagship species for the creation of new nature reserves in Madagascar. *Salamandra* **46**, 167–179 (2010).
Dogania subplana	[4] Malkmus, R., Manthey, U., Vogel, G., Hoffmann, P. & Kosuch, J. *Amphibians and Reptiles of Mount Kinabalu (North Borneo)*. (2002)
Leiopython meridionalis	[5] Natusch, D. & Lyons, J. Ecological attributes and trade of white-lipped pythons (Genus Leiopython) in Indonesian New Guinea. *Aust. J. Zool.* **59**, 339–343 (2012).
Micrurus nigrocinctus	[6] Solórzano, A. & Cerdas, L. [Reproductive cycles of the coral snake Micrurus nigrocinctus (Serpentes: Elapidae) in Costa Rica]. *Rev. Biol. Trop.* **36**, 235–239 (1988).
**Maximum longevity**	
Cerberus rynchops	[4] Malkmus, R., Manthey, U., Vogel, G., Hoffmann, P. & Kosuch, J. *Amphibians and Reptiles of Mount Kinabalu (North Borneo)*. (2002)
Chitra indica	[7] Hanus, K. Chitra indica (Narrow-headed soft-shelled Turtle). *Animal Diversity Web* https://animaldiversity.org/accounts/Chitra_indica/.
Chitra vandijki	[8] Trionychidae (28 Different Species) - All Turtles. https://www.allturtles.com/trionychidae/ (2022).
Cyclemys pulchristriata	[9] Geoemydidae (56 Different Species) - All Turtles. https://www.allturtles.com/geoemydidae/ (2022).
Cycloderma frenatum	[8] Trionychidae (28 Different Species) - All Turtles. https://www.allturtles.com/trionychidae/ (2022).
Kinyongia uthmoelleri	[10] Lutzmann, N. *et al.* Description of a new subspecies of Kinyongia uthmoelleri (Müller, 1938) (Squamata: Chamaeleonidae) with notes on its captive propagation. *Bonn Zool. Bull.* **57**, 281–288 (2010).

Phelsuma robertmeitensi	[11] Stark, G., Pincheira-Donoso, D. & Meiri, S. No evidence for the 'rate-of-living' theory across the tetrapod tree of life. *Glob. Ecol. Biogeogr.* **29**, 857–884 (2020).
Pyxis arachnoides	[12] CONVENTION ON INTERNATIONAL TRADE IN ENDANGERED SPECIES. *Transfer of Pyxis arachnoides from Appendix II to Appendix I, in accordance with Resolution Conf. 9.24 (Rev. CoP12), Annex 1, paragraphs B. i), iii) and iv), and C. i).* https://cites.org/sites/default/files/eng/cop/13/prop/E13-P15.pdf (2004).
Rhamphoeleon spectrum	[13] Tolley, K. *Chameleons of Southern Africa*. (Penguin Random House South Africa, 2012).

Appendix S1: Table S3

Excerpt of the importer-country trade matrix, containing the number of specimens of each species imported by the respective countries.

**Importer Country**	**Number of specimens of species traded**			
	Varanus.indicus	Alligator.mississippiensis	Caiman.crocodilus	Calabaria.reinhardtii
Bolivia	0	189	15100	0
Brazil	2	0	3005	0
Bulgaria	0	0	0	0
Canada	52	670	3162	647

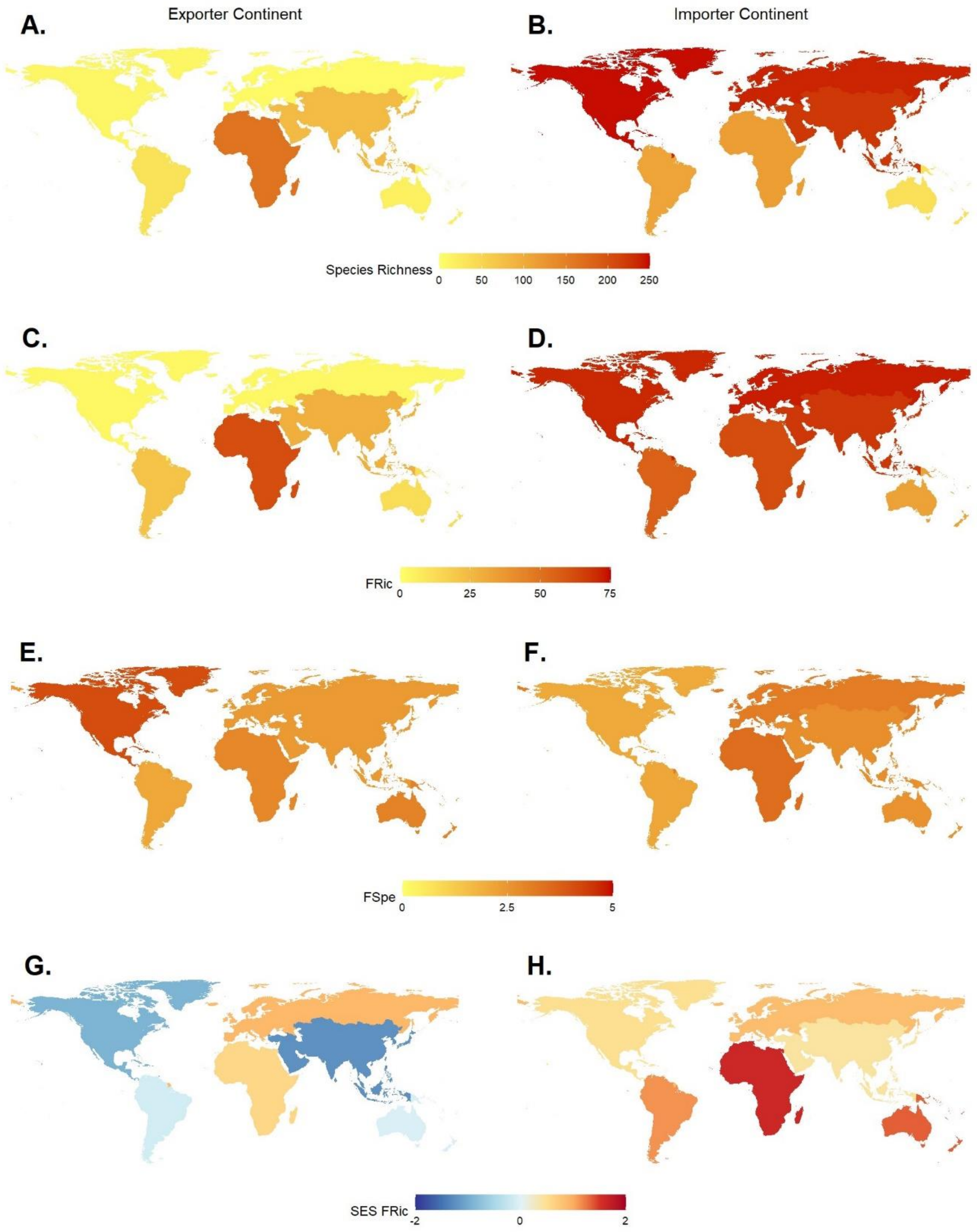

Appendix S1: Figure S1. Functional composition of continental trade assemblage. Species richness values of continental **A)** exported and **B)** imported assemblages. FRic values of continental **C)** exported and **D)** imported assemblages. FSpe values of continental **E)** exported and **F)** imported assemblages. SES FRic values of continental **G)** exported and **H)** imported assemblages.

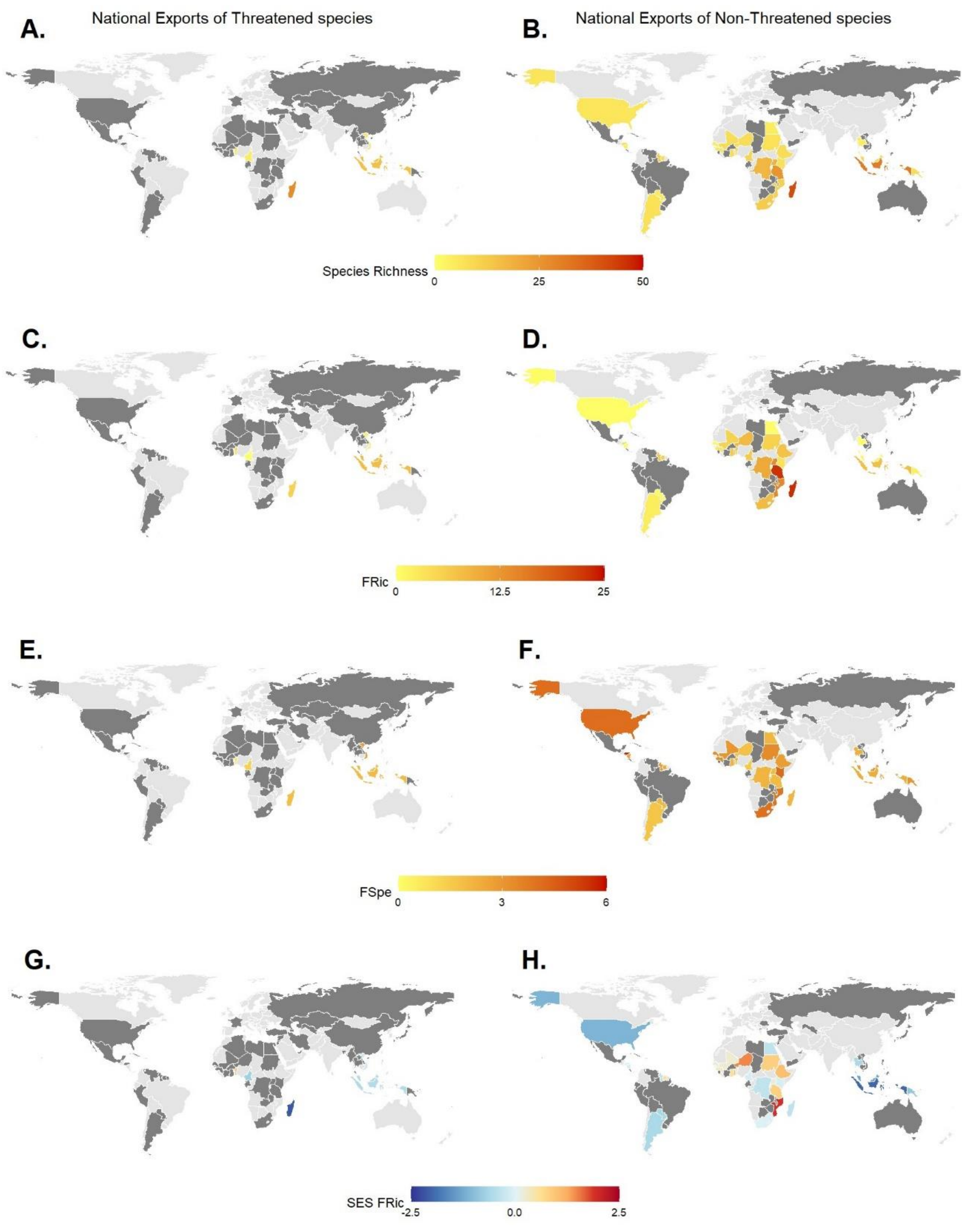

Appendix S1: Figure S2. Functional composition of national exported trade assemblages of IUCN classified threatened and non-threatened species. Species richness values of national **A)** threatened and **B)** non-threatened assemblages. FRic values of national **C)** threatened and **D)** non-threatened assemblages. FSpe values of national **E)** threatened and **F)** non-threatened assemblages. SES FRic values of national **G)** threatened and **H)** non-threatened assemblages. Countries shaded in light grey did not document any trade between 2000-2020, whilst countries shaded in dark grey did not export enough species to generate functional metrics.

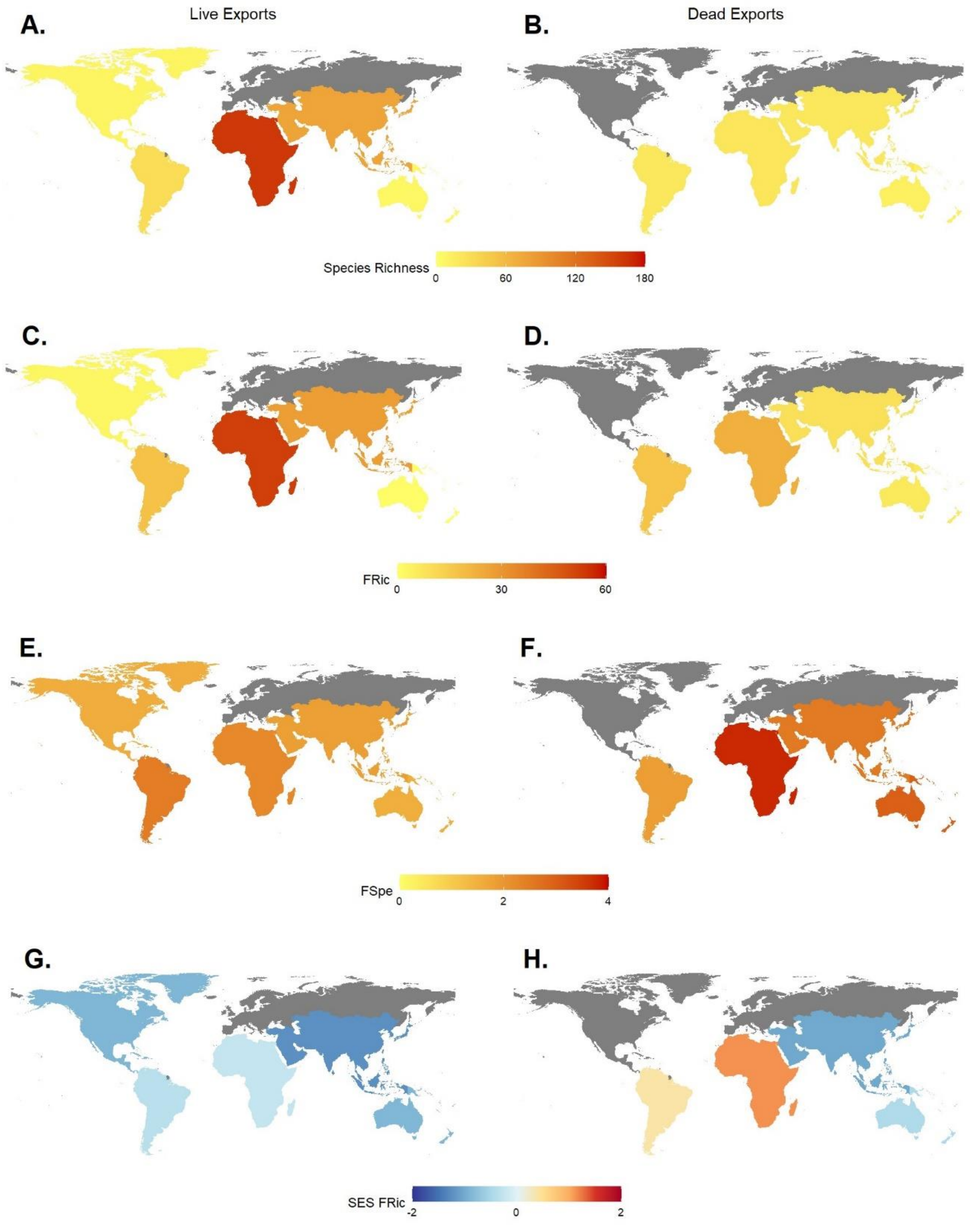

Appendix S1: Figure S3. Functional composition of live and dead exported trade assemblages Species richness values of continental **A)** live and **B)** dead assemblages. FRic values of continental **C)** live and **D)** dead assemblages. FSpe values of continental **E)** live and **F)** dead assemblages. SES FRic values of continental **G)** live and **H)** dead assemblages.

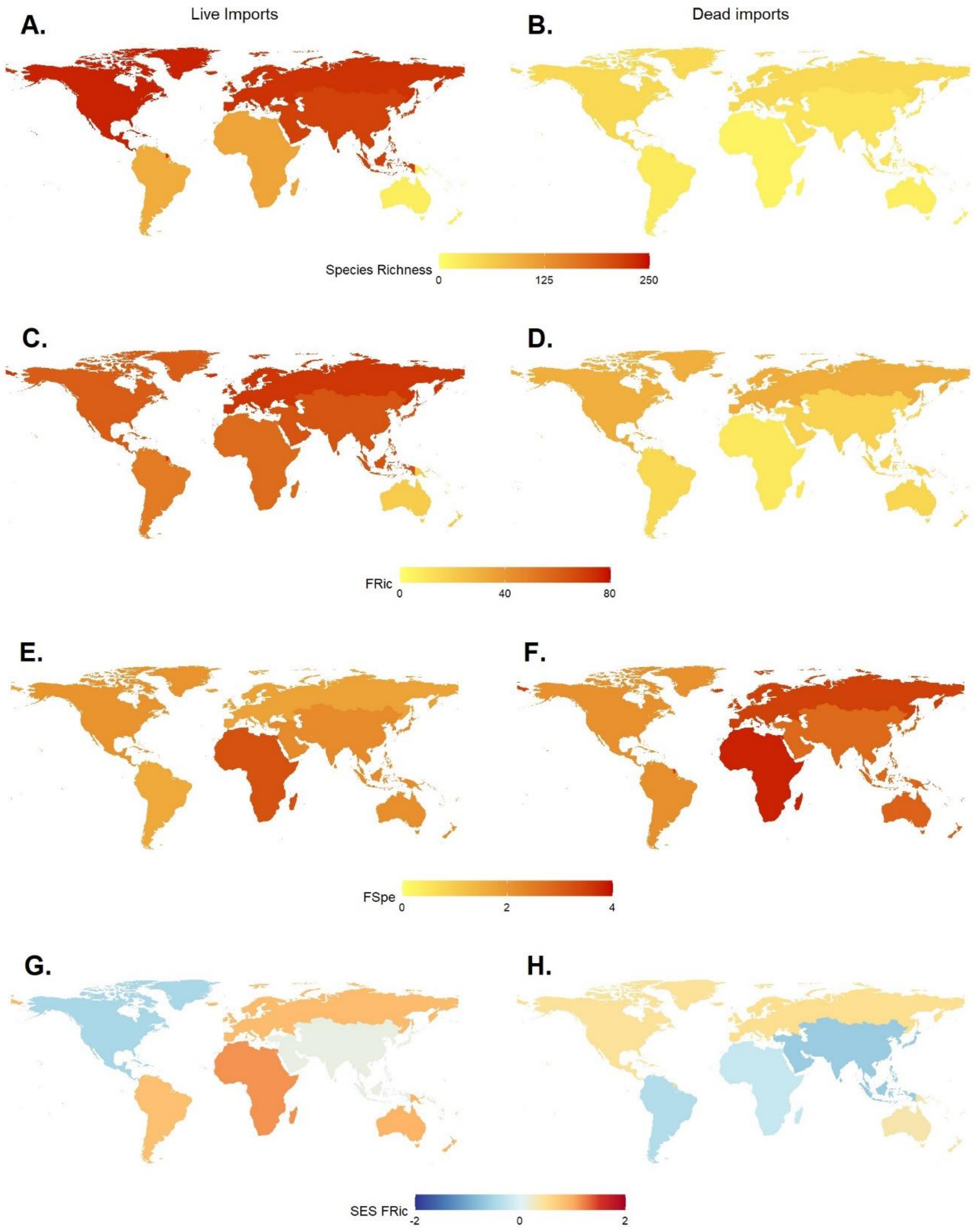

Appendix S1: Figure S4. Functional composition of live and dead imported trade assemblages Species richness values of continental **A)** live and **B)** dead assemblages. FRic values of continental **C)** live and **D)** dead assemblages. FSpe values of continental **E)** live and **F)** dead assemblages. SES FRic values of continental **G)** live and **H)** dead assemblages.

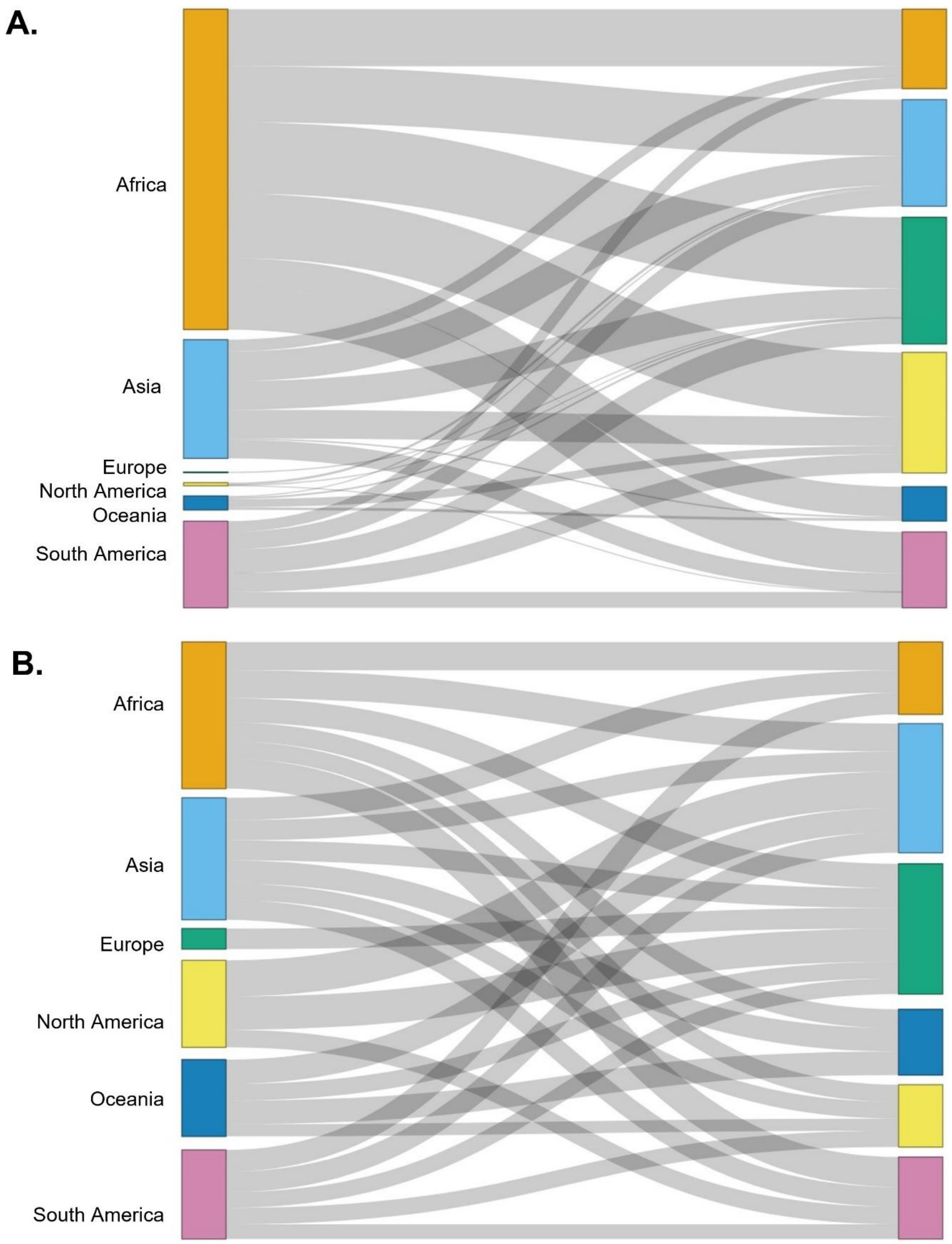

Appendix S1: Figure S5: Functional richness of continental trade routes. A) FRic and **B)** FSpe values associated with the traded assemblages of continental trade routes. Linewidth represents the relative value of each functional metric associated with the individual trade routes. Node size represents the cumulative FRic, FSpe or SES FRic value associated with all route assemblages of the specified exporting or importing continent.

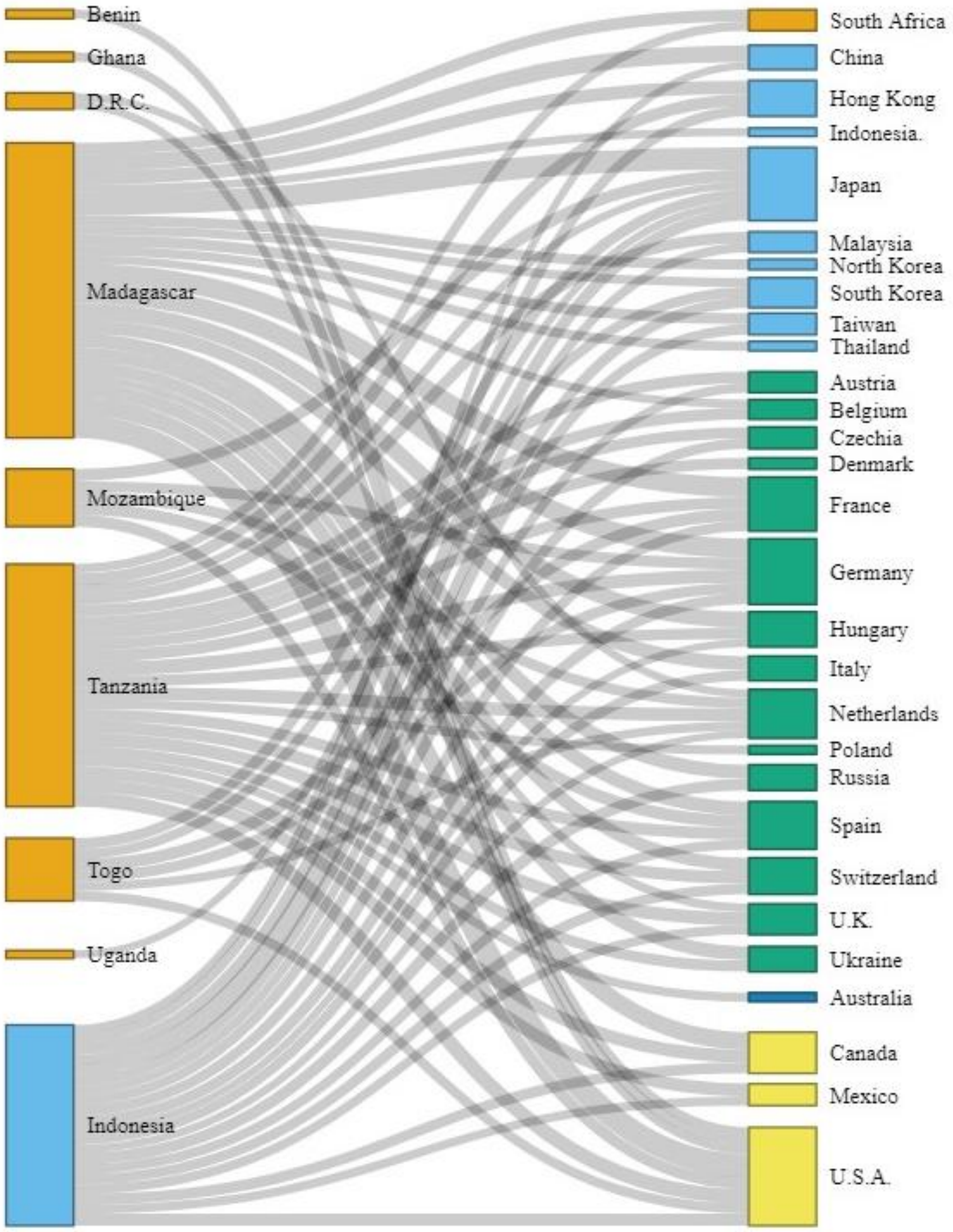

Appendix S1: Figure S6: Functional richness of key national trade routes. Linewidth represents the FRic value associated with the individual trade routes. Node size represents the cumulative FRic value associated with all route assemblages of the specified exporting or importing country. Route assemblages with FRic values lesser than 10 were excluded from the plot.

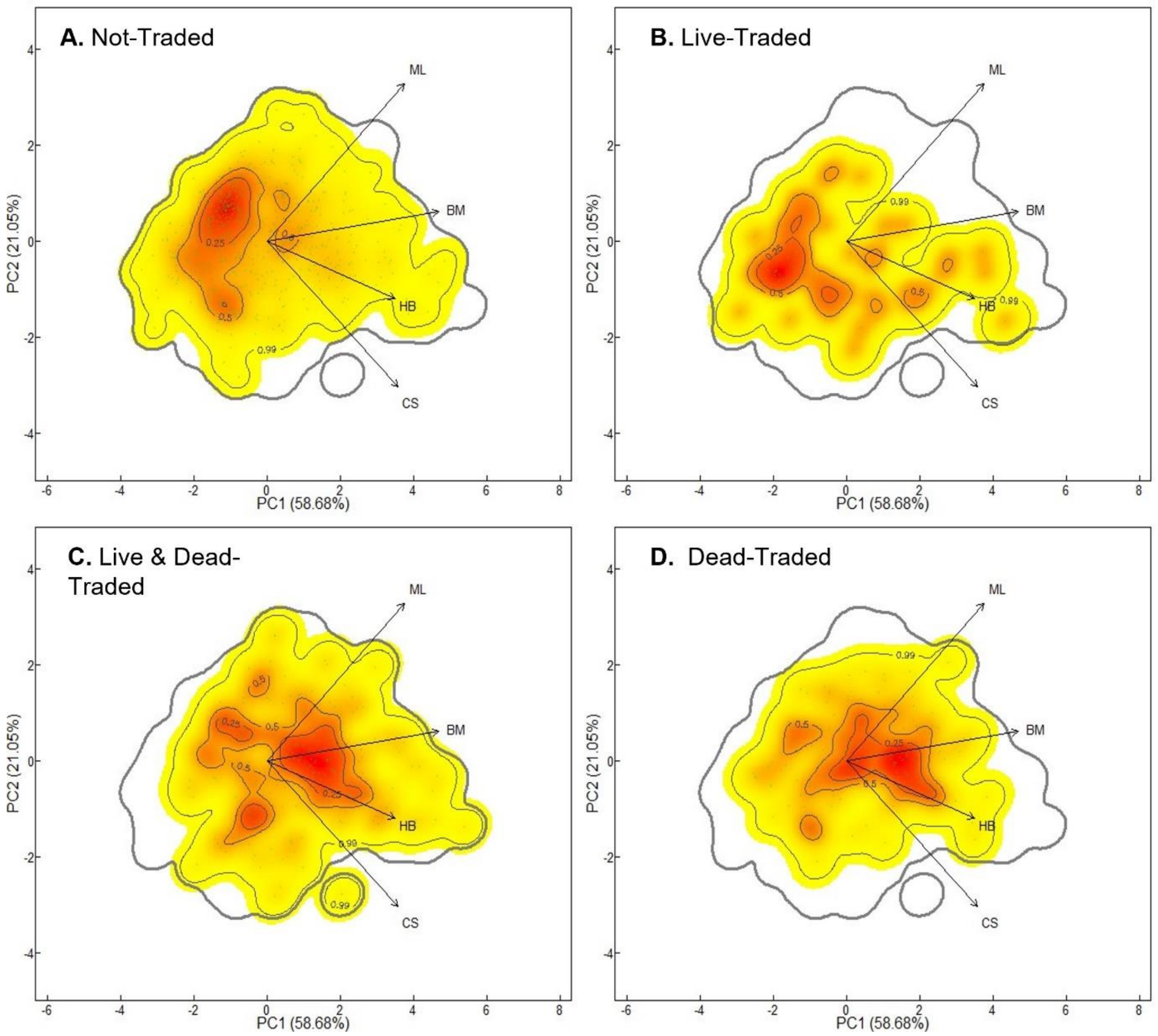

Appendix S1: Figure S7. The probabilistic species distribution of **A)** non-traded, **B)** wild-only traded, **C)** wild and captive traded, and **D)** captive-only traded assemblages within the functional space of all CITES listed species, defined by the two first principal components axes (PC1 = 58.68% and PC2 = 21.05% of variance explained) of a principal component analysis (PCA). The arrows indicate the direction and weighting of the functional traits (body mass (BM), clutch size (CS), maximum longevity (ML), habitat breadth (HB)) in the PCA. The colour gradient (red, orange, and yellow) depicts the density of species in the functional space, where red corresponds to more densely populated areas. Thick contour lines indicate the outer limits of the functional space and thinner lines indicate quantiles 0.25, 0.5, 0.99.

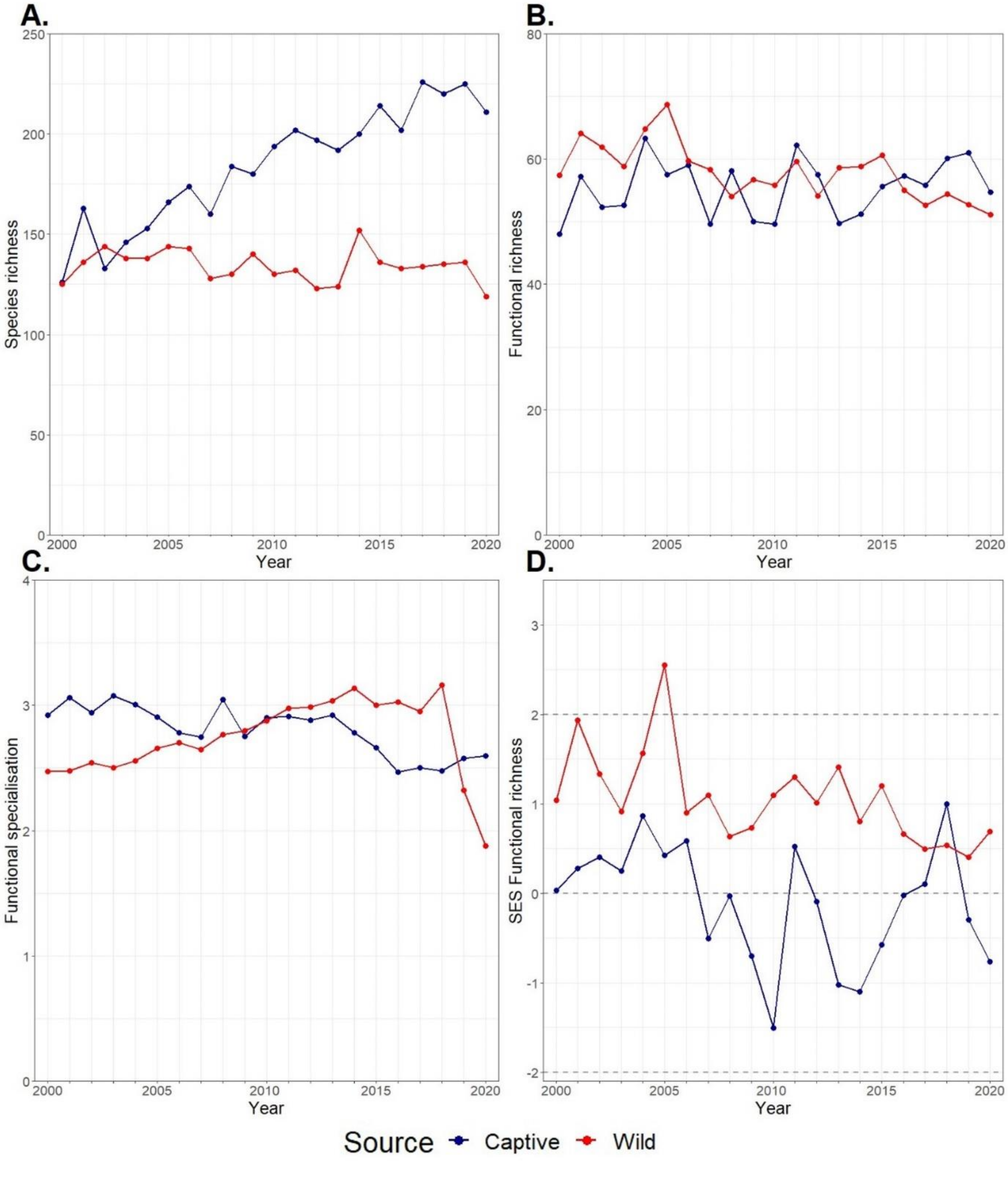

Appendix S1: Figure S8. Functional composition of captive and wild-sourced trade assemblages. **A)** Species richness, **B)** FRic, **C)** FSpe, and **D)** SES Fric values of captive-bred and wild-sourced total traded assemblages from 2000-2020.

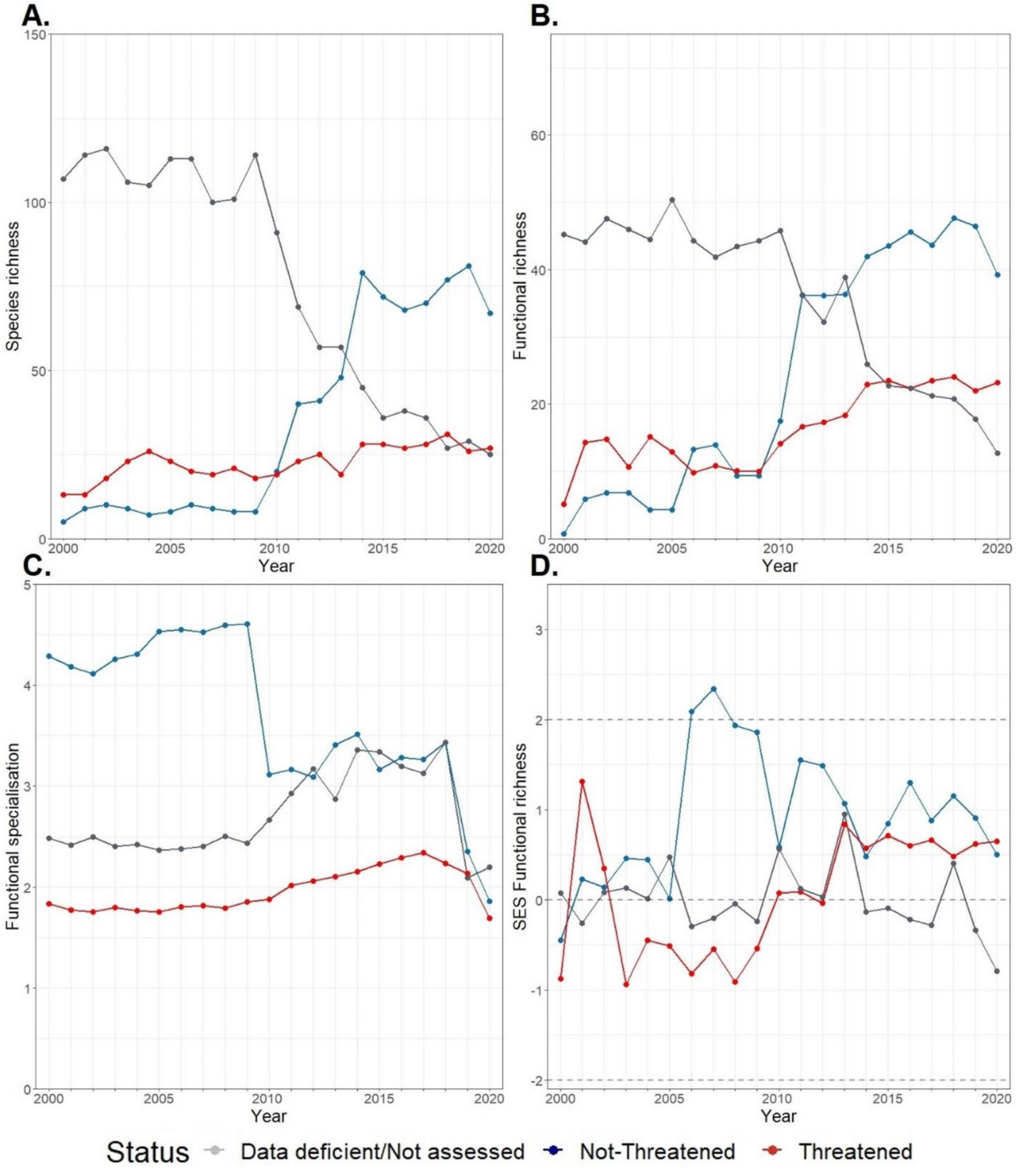

Appendix S1: Figure S9. Functional composition of threatened, non-threatened, and data deficient trade assemblages. A) Species richness, **B)** FRic, **C)** FSpe, and **D)** SES values of threatened, non-threatened, and data deficient/non-assessed traded assemblages from 2000-2020.

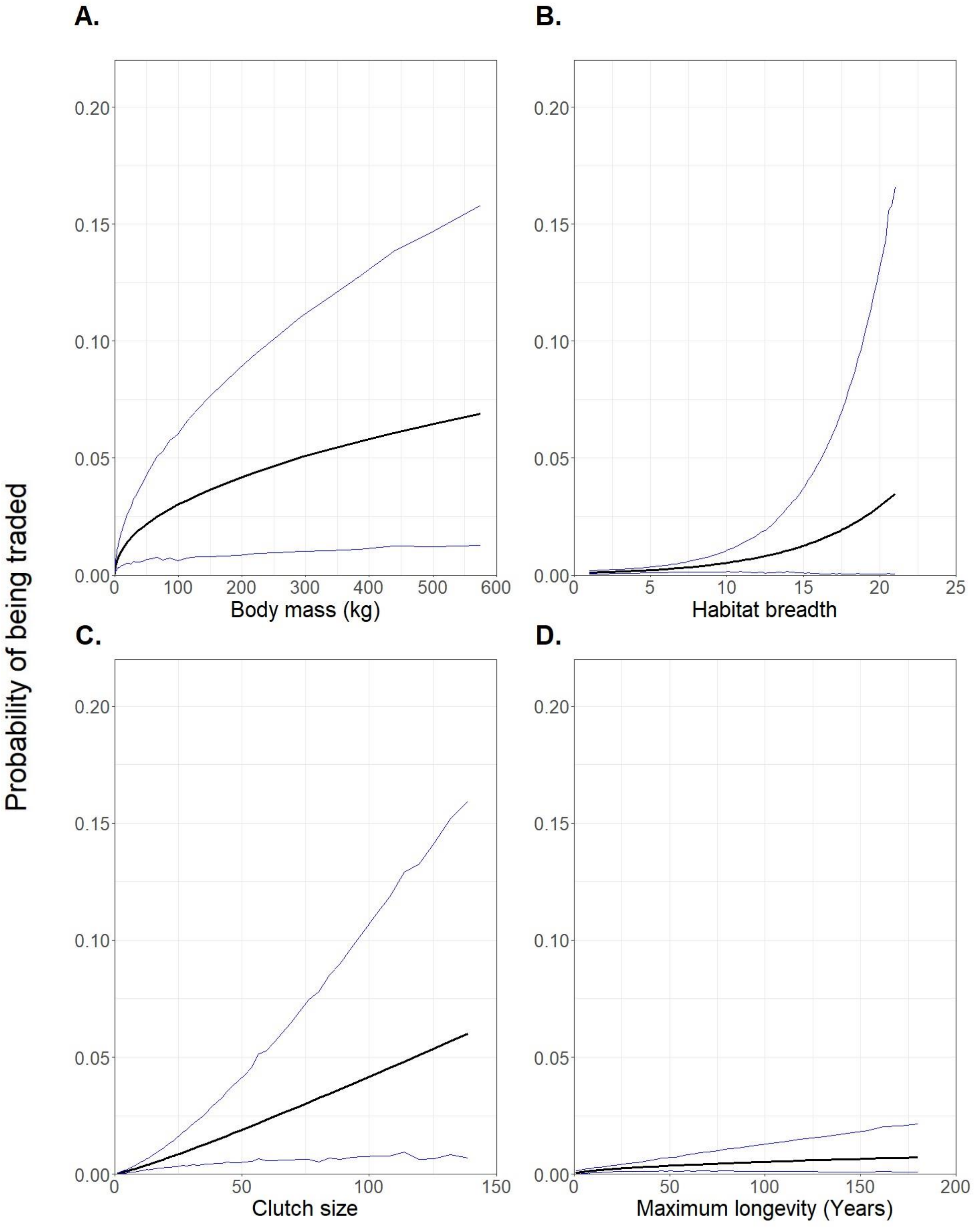

Appendix S1: Figure S10. Global associations between functional traits and the probability of being traded. A) body mass, **B)** habitat breadth, **C)** clutch size and **D)** maximum longevity. The black line represents the posterior medians, and the blue lines represent the and upper and lower 90% HDCI bounds.

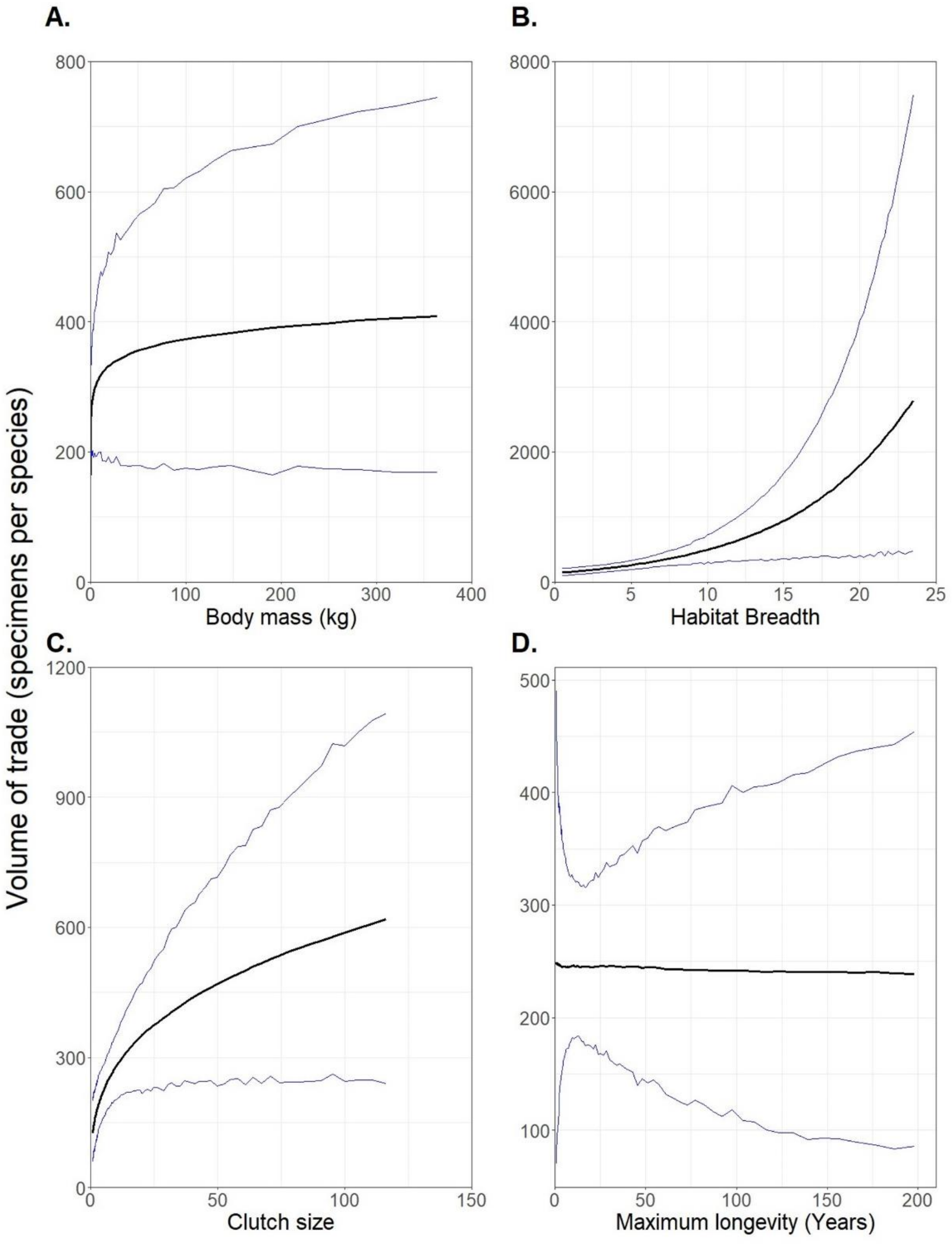

Appendix S1: Figure S11. Global associations between functional traits and the volume of trade. A) body mass, **B)** habitat breadth, **C)** clutch size and **D)** maximum longevity. The black line represents the posterior medians, and the blue lines represent the and upper and lower 90% HDCI bounds.

Appendix S1: Table S4. Model estimates for the change in the log odds of being traded associated with increases in the magnitude of functional traits among all traded CITES-listed species. Estimates across the whole posterior to the median and upper and lower 90% HDCI bounds. Pd denotes the direction probability of direction, values greater than 97.50% provide high certainty of directional effect, whilst values at 50% mean the posterior is evenly split between positive and negative estimates.

**Parameter**	**Median**	**Lower**	**Upper**	***Pd* (%)**
Body mass	1.24	0.83	1.60	**100.00**
Clutch size	2.84	1.80	3.90	**100.00**
Habitat breadth	0.52	0.39	0.67	**100.00**
Maximum longevity	0.836	-0.188	2.01	90.00

Appendix S1: Table S5. Model estimates for percentage change in trade volume associated with increases in the magnitude of functional traits among all traded CITES-listed species. Estimates across the whole posterior to the median and upper and lower 90% HDCI bounds.

**Parameter**	**Median**	**Lower**	**Upper**	***Pd* (%)**
Body mass	1.22	0.96	1.49	91.70
Clutch size	2.28	1.04	3.48	**98.80**
Habitat breadth	1.16	1.07	1.25	**99.70**
Maximum longevity	1.15	0.43	1.87	55.40

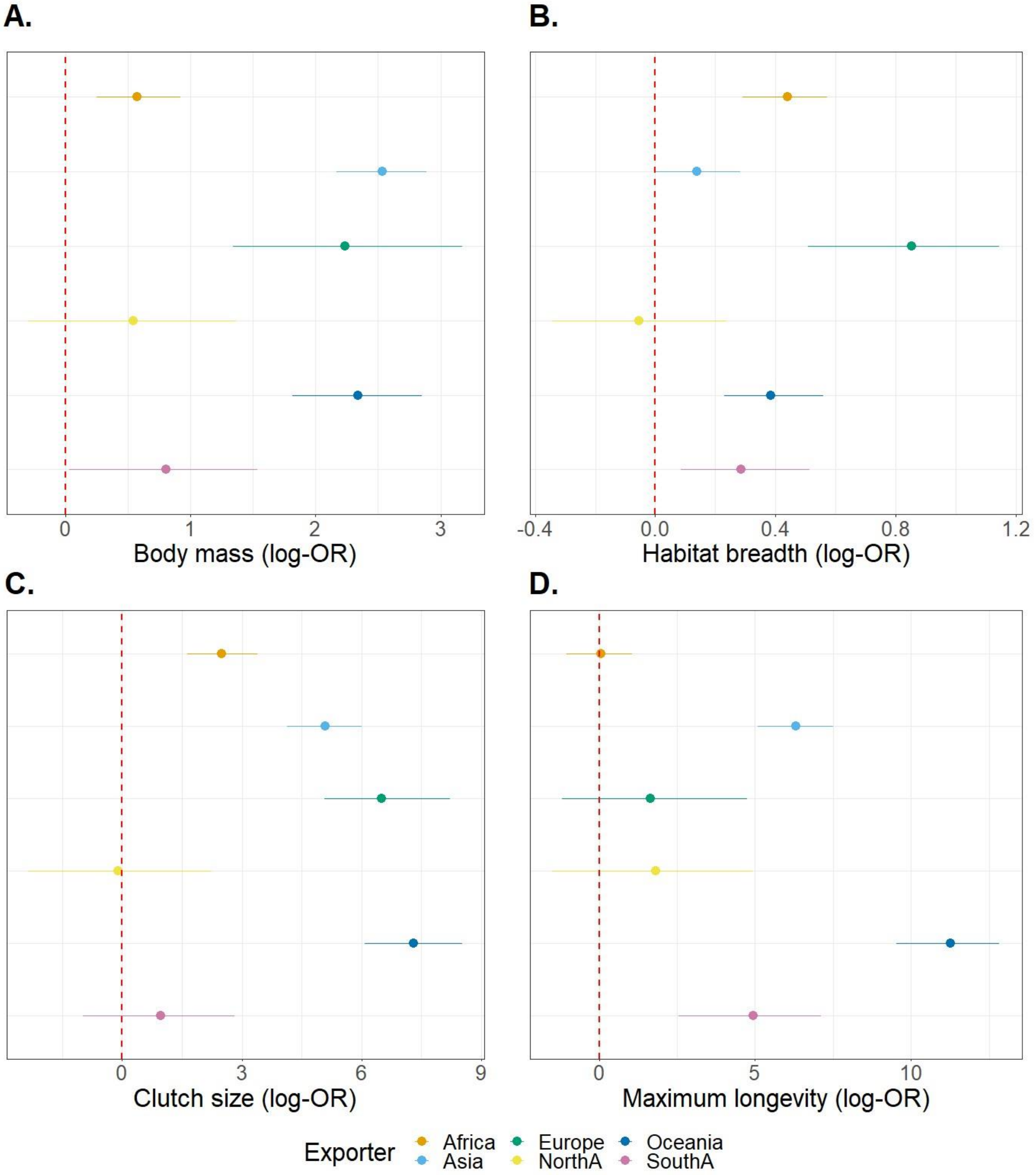

Appendix S1: Figure S12. The change in log odds of being traded resulting from increases in the magnitude of functional traits in continental exported assemblages. **A)** body mass **B)** clutch size, **C)** habitat breadth and **D)** maximum longevity. Points represent posterior medians, and error bars represent the and upper and lower 90% HDCI bounds.

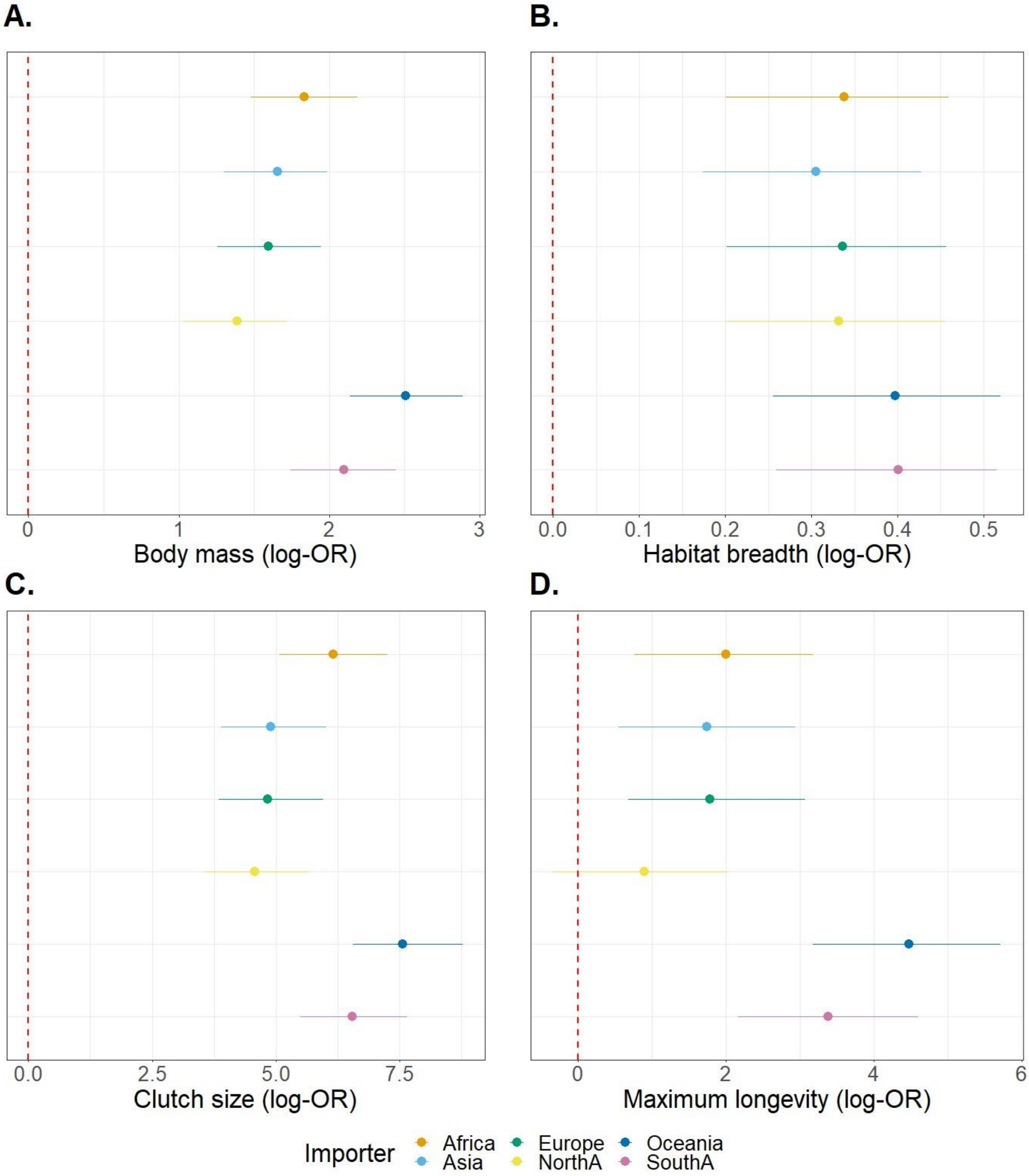

Appendix S1: Figure S13. The change in log odds of being traded resulting from increases in the magnitude of functional traits in continental imported assemblages. **A)** body mass **B)** clutch size, **C)** habitat breadth and **D)** maximum longevity. Points represent posterior medians, and error bars represent the and upper and lower 90% HDCI bounds.

Appendix S1: Table S6. Model estimates for the change in the log odds of being traded associated with increases in the magnitude of functional traits within continental exported assemblages.

Estimates across the whole posterior to the median and upper and lower 90% HDCI bounds.

Continent	Parameter	Median	Lower	Upper	*Pd* (%)
Africa	Body mass	0.57	0.25	0.92	**99.80**
	Clutch size	2.50	1.64	3.40	**100.00**
	Habitat breadth	0.44	0.29	0.57	**100.00**
	Maximum longevity	0.06	-1.03	1.07	54.20
Asia	Body mass	2.53	2.17	2.89	**100.00**
	Clutch size	5.09	4.14	6.02	**100.00**
	Habitat breadth	0.14	0.002	0.28	94.70
	Maximum longevity	6.31	5.10	7.51	**100.00**
Europe	Body mass	2.23	1.34	3.17	**100.00**
	Clutch size	6.50	5.06	8.23	**100.00**
	Habitat breadth	0.85	0.51	1.14	**100.00**
	Maximum longevity	1.65	-1.20	4.74	81.40
North America	Body mass	0.54	-0.29	1.37	86.80
	Clutch size	-0.11	-2.35	2.25	52.00
	Habitat breadth	-0.05	-0.34	0.24	63.00
	Maximum longevity	1.82	-1.49	4.92	82.10
Oceania	Body mass	2.34	1.81	2.85	**100.00**
	Clutch size	7.32	6.08	8.53	**100.00**
	Habitat breadth	0.39	0.23	0.56	**100.00**
	Maximum longevity	11.30	9.53	12.80	**100.00**
South America	Body mass	0.81	0.03	1.54	96.20
	Clutch size	0.97	-0.98	2.82	79.10
	Habitat breadth	0.29	0.08	0.51	**99.80**
	Maximum longevity	4.94	2.56	7.11	**100.00**

Appendix S1: Table S7. Model estimates for the change in the log odds of being traded associated with increases in the magnitude of functional traits within continental imported assemblages. Estimates across the whole posterior to the median and upper and lower 90% HDCI bounds.

Continent	Parameter	Median	Lower	Upper	*Pd* (%)
Africa	Body mass	1.84	1.48	2.19	**100.00**
	Clutch size	6.16	5.08	7.25	**100.00**
	Habitat breadth	0.34	0.20	0.46	**100.00**
	Maximum longevity	2.00	0.76	3.18	**99.80**
Asia	Body mass	1.65	1.30	1.98	**100.00**
	Clutch size	4.90	3.89	6.02	**100.00**
	Habitat breadth	0.31	0.18	0.43	**100.00**
	Maximum longevity	1.75	0.55	2.94	**99.40**
Europe	Body mass	1.59	1.25	1.94	**100.00**
	Clutch size	4.84	3.85	5.96	**100.00**
	Habitat breadth	0.34	0.20	0.46	**100.00**
	Maximum longevity	1.78	0.68	3.08	**99.50**
North America	Body mass	1.39	1.03	1.72	**100.00**
	Clutch size	4.57	3.55	5.68	**100.00**
	Habitat breadth	0.33	0.20	0.45	**100.00**
	Maximum longevity	0.90	-0.34	2.02	89.20
Oceania	Body mass	2.51	2.14.	2.89	**100.00**
	Clutch size	7.57	6.56	8.78	**100.00**
	Habitat breadth	0.40	0.26	0.52	**100.00**
	Maximum longevity	4.47	3.17	5.72	**100.00**
South America	Body mass	2.10	1.74	2.44	**100.00**
	Clutch size	6.54	5.49	7.65	**100.00**
	Habitat breadth	0.40	0.30	0.52	**100.00**
	Maximum longevity	3.38	2.16	4.60	**100.00**

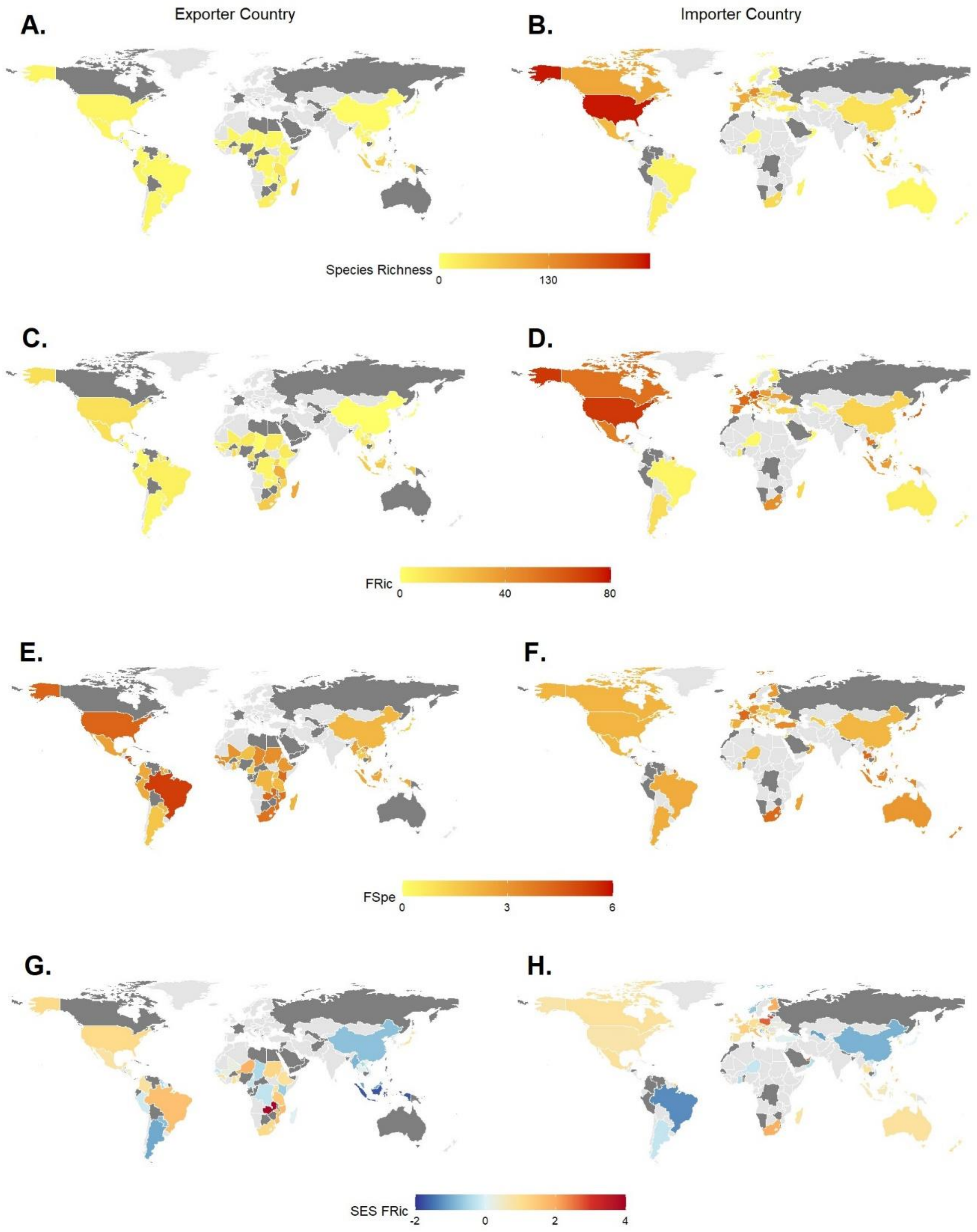

Appendix S1: Figure S14. Replication of Figure 1 using importer-reported CITES trade data (rather than exporter-reported).

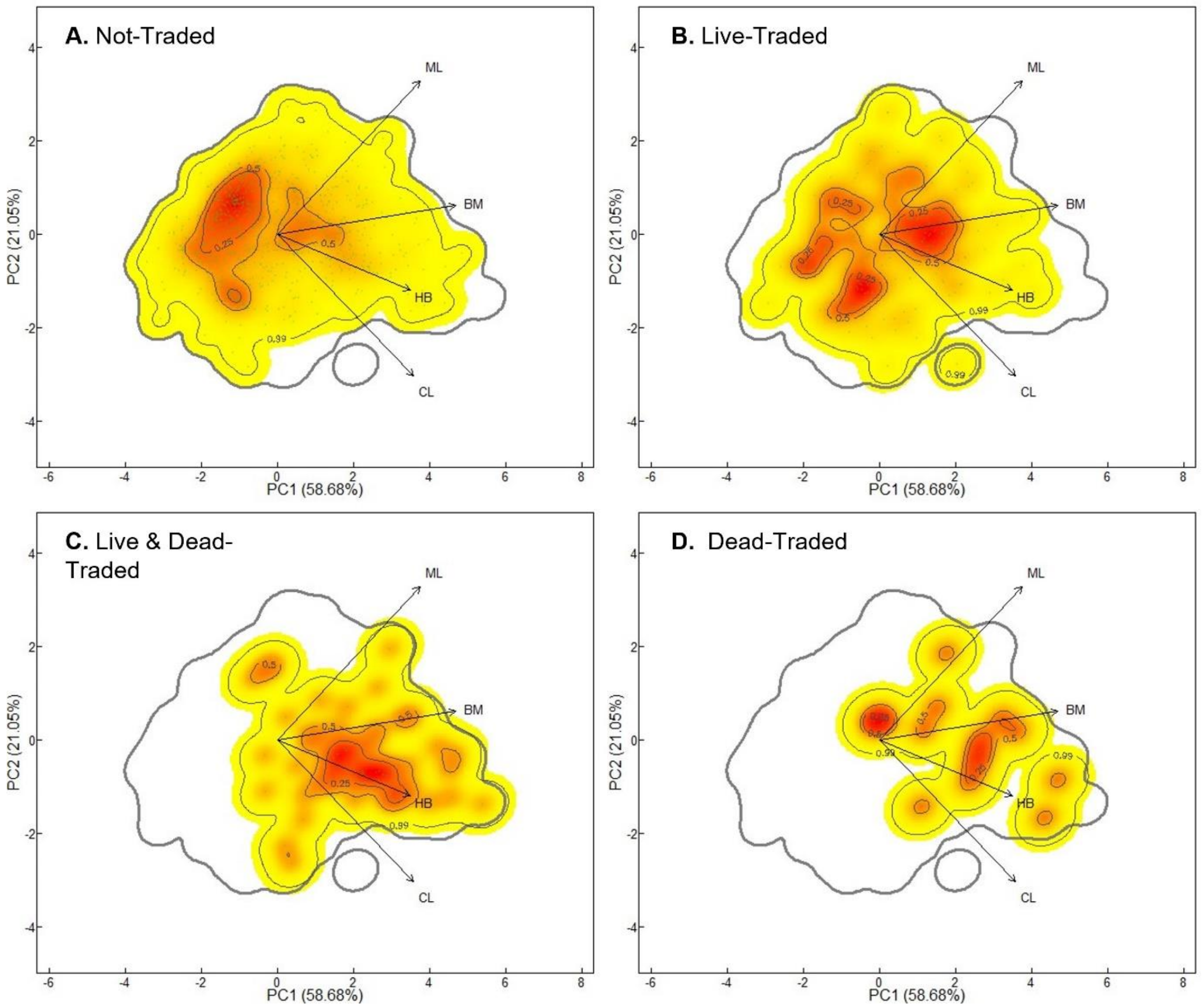

Appendix S1: Figure S15. Replication of Figure 2 using importer-reported CITES trade data (rather than exporter-reported).

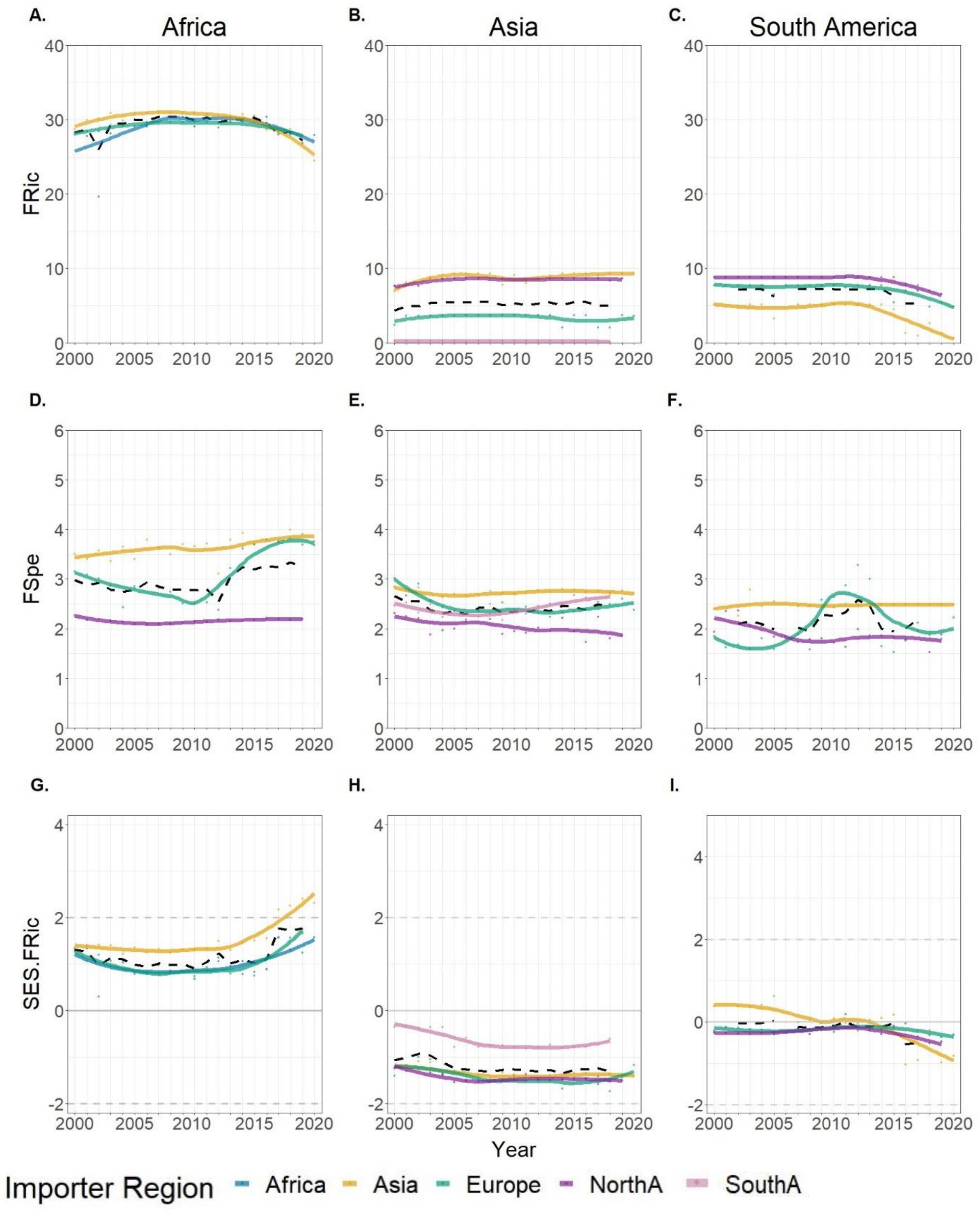

Appendix S1: Figure S16. Replication of Figure 3 using importer-reported CITES trade data (rather than exporter-reported).

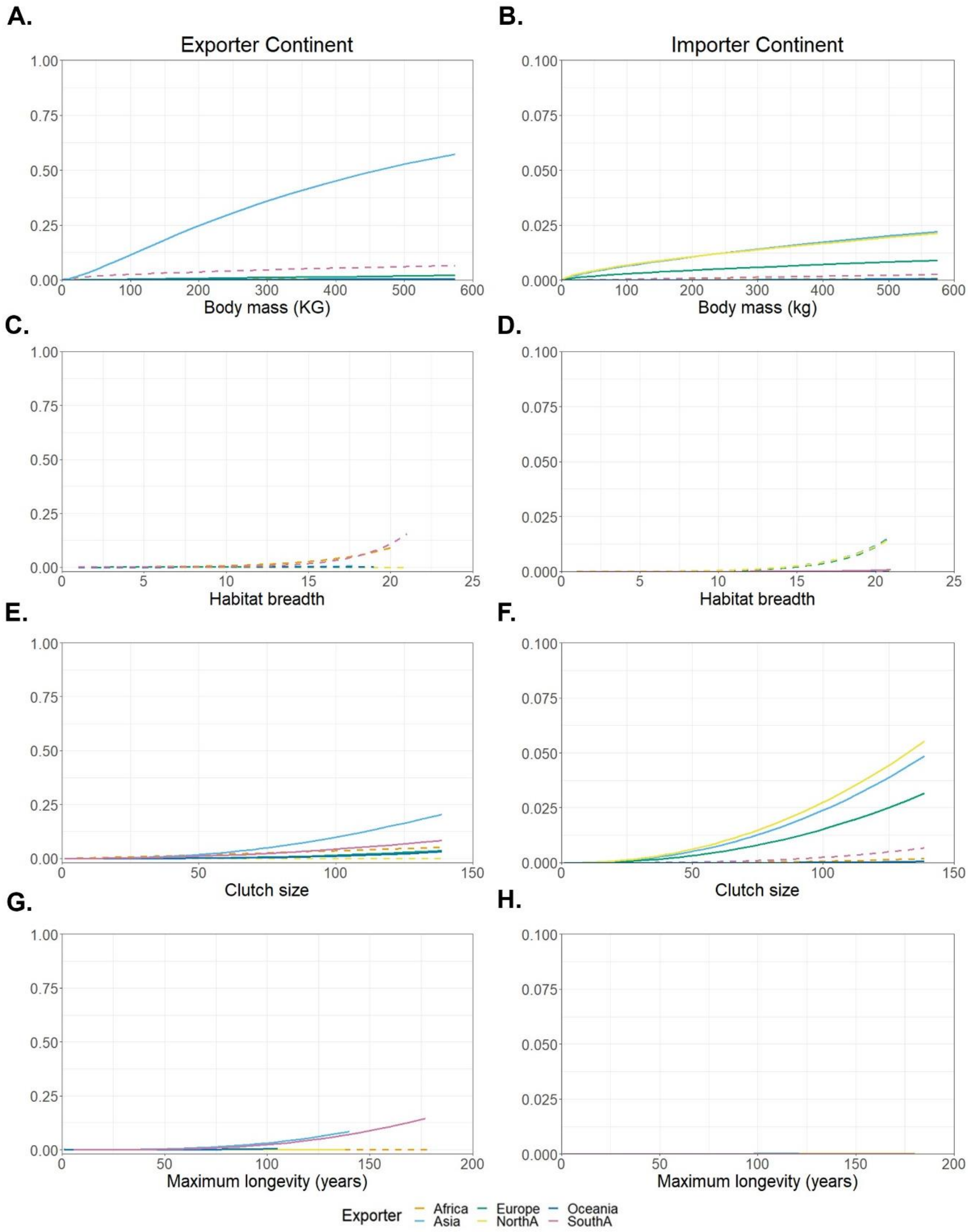

Appendix S1: Figure S17. Replication of Figure 4 using importer-reported CITES trade data (rather than exporter-reported).

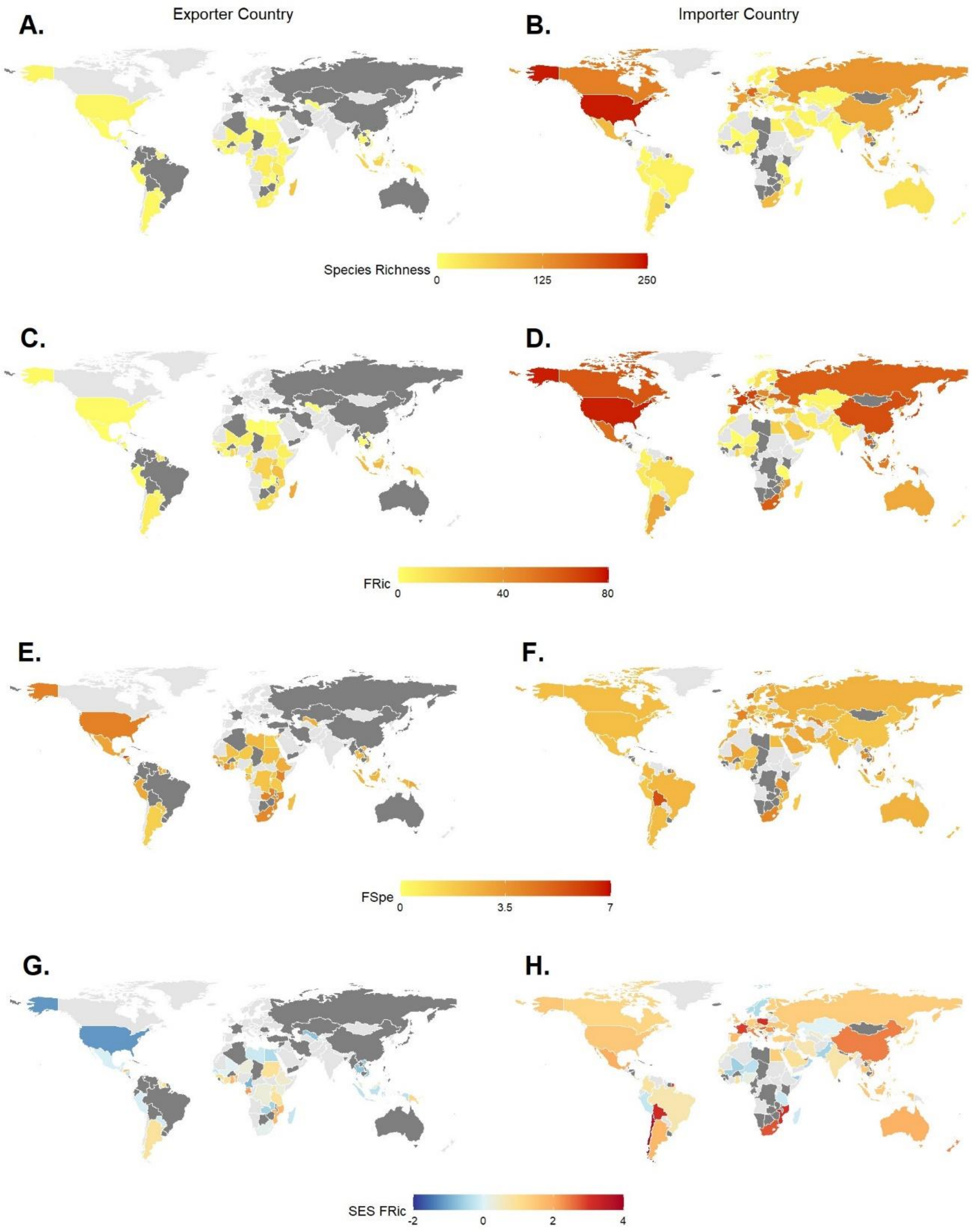

Appendix S1: Figure S18. Replication of Figure 1 using an alternative dataset from the MICE imputation for missing functional trait values.

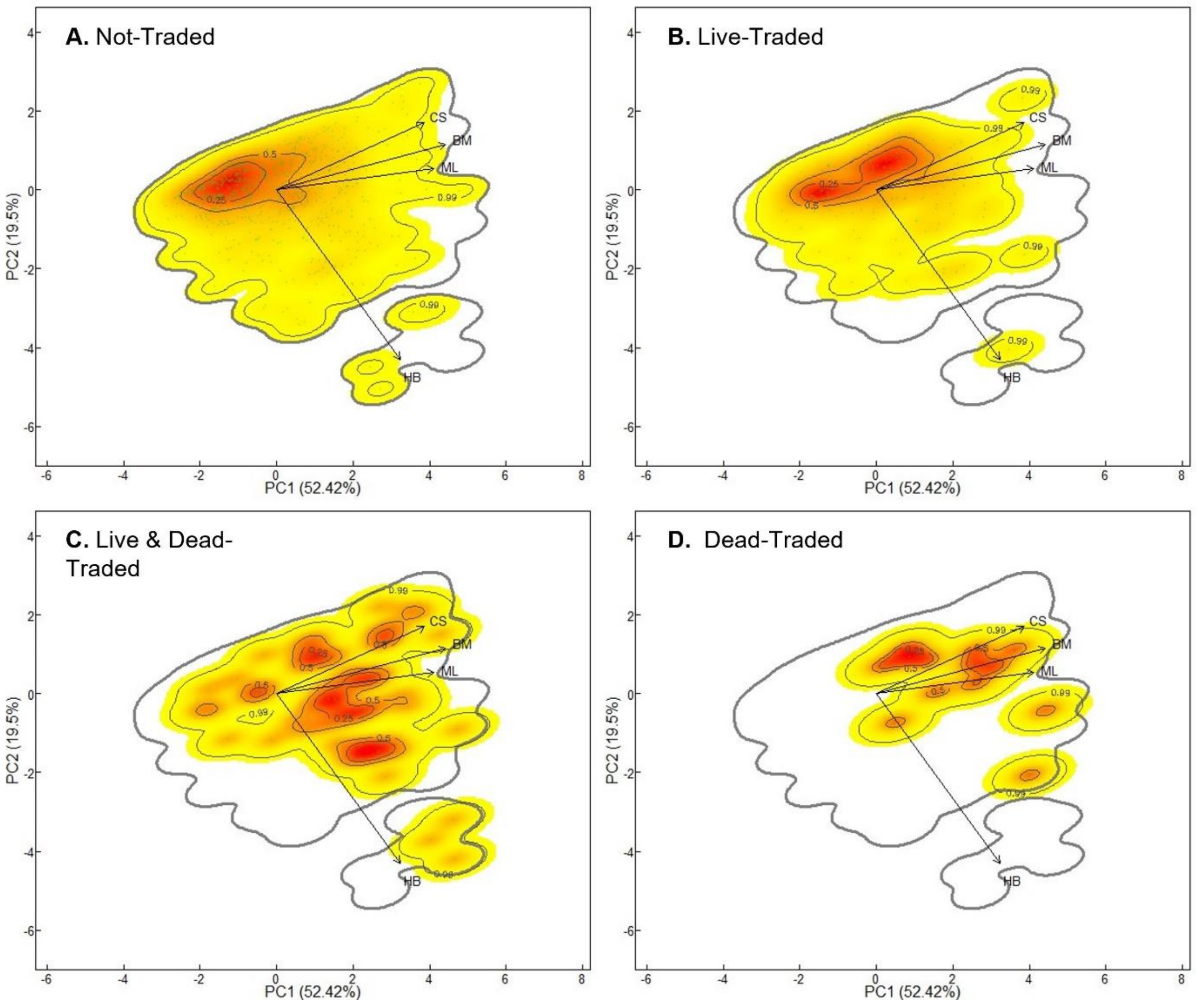

Appendix S1: Figure S19. Replication of Figure 2 using an alternative output from the MICE imputation for missing functional trait values.

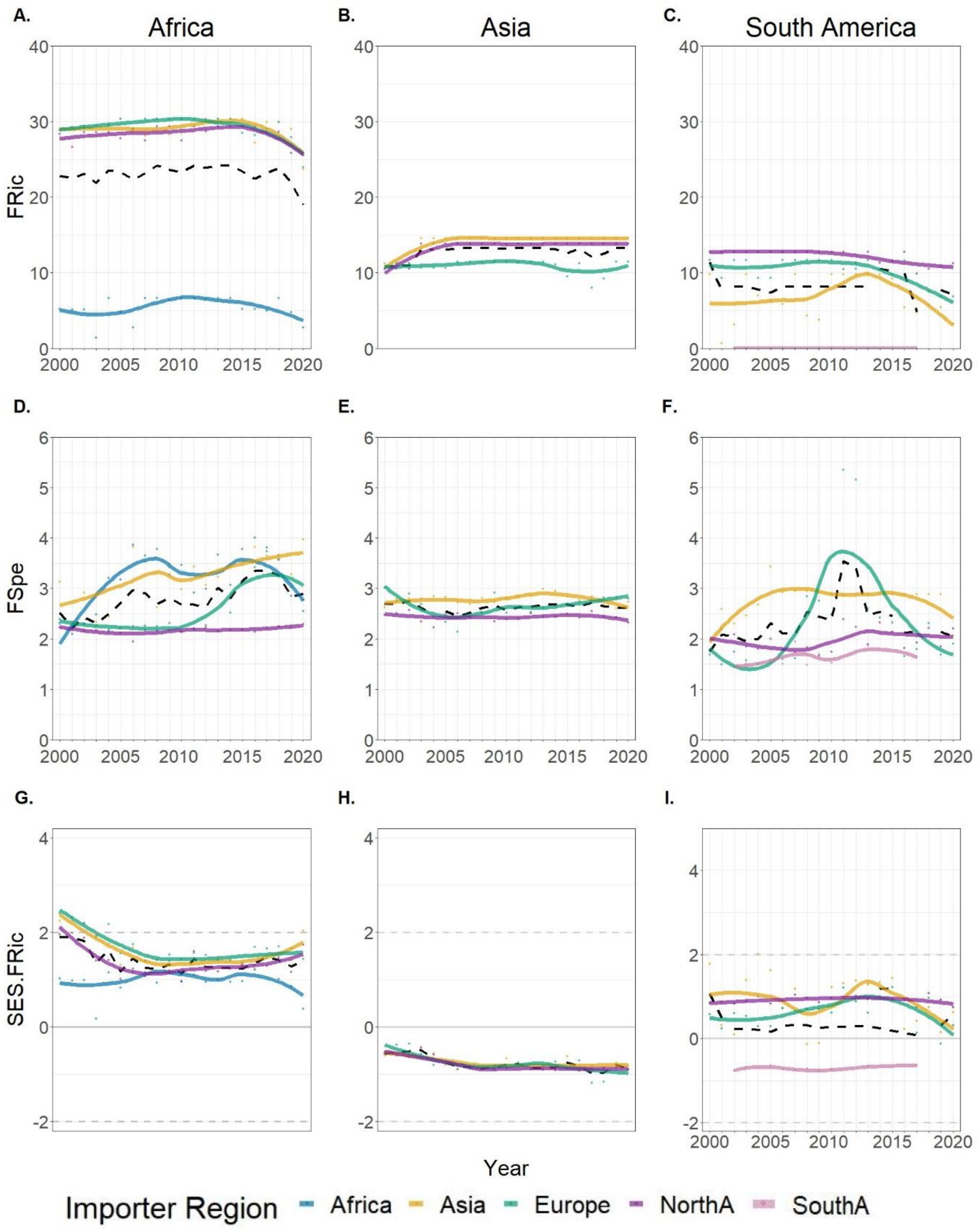

Appendix S1: Figure S20. Replication of Figure 3 using an alternative output from the MICE imputation for missing functional trait values.

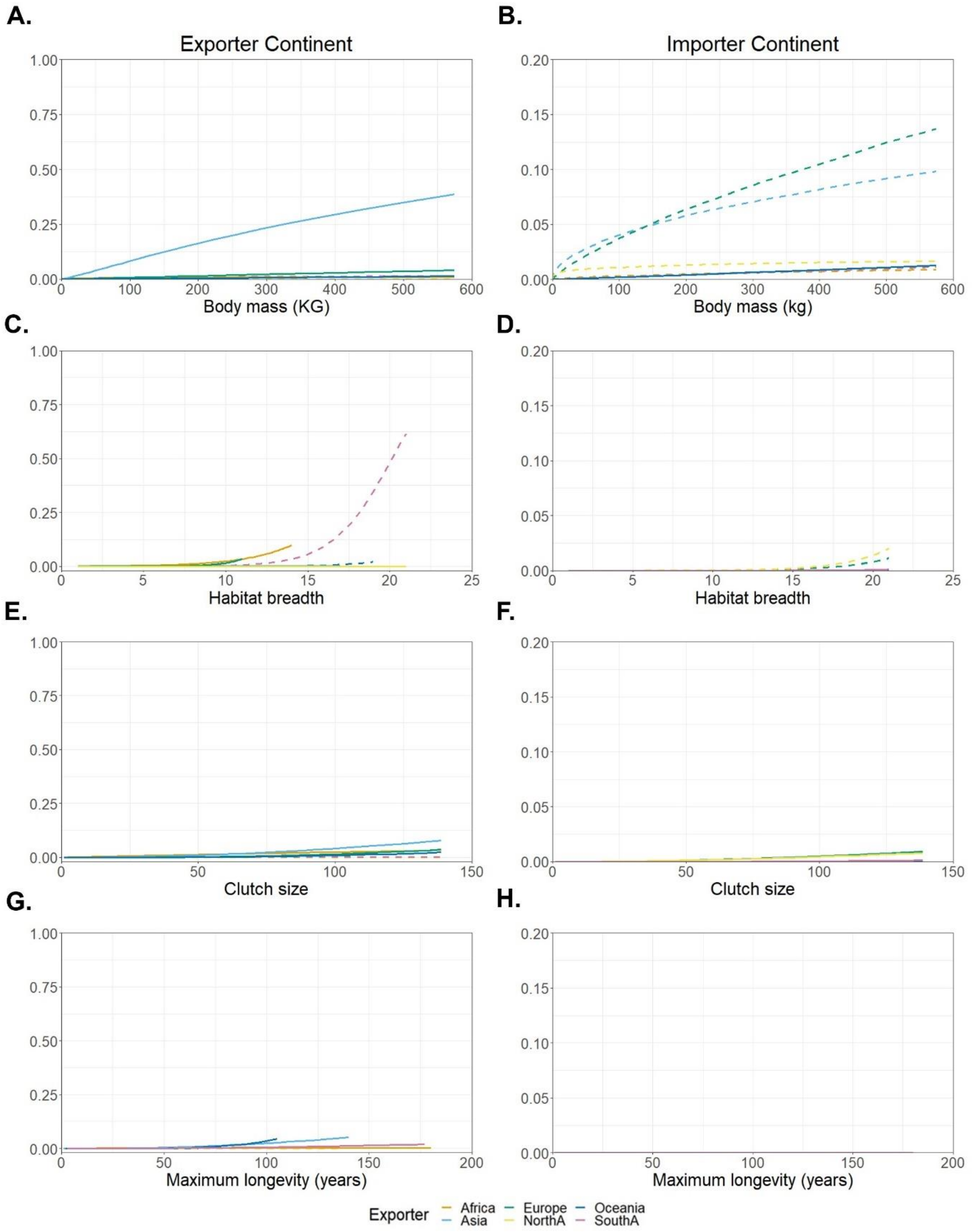

Appendix S1: Figure S21. Replication of Figure 4 using an alternative output from the MICE imputation for missing functional trait values.
